# Transactivation specificity is conserved among p53 family proteins and depends on a response element sequence code

**DOI:** 10.1093/nar/gkt657

**Published:** 2013-07-26

**Authors:** Yari Ciribilli, Paola Monti, Alessandra Bisio, H. Thien Nguyen, Abdul S. Ethayathulla, Ana Ramos, Giorgia Foggetti, Paola Menichini, Daniel Menendez, Michael A. Resnick, Hector Viadiu, Gilberto Fronza, Alberto Inga

**Affiliations:** ^1^Laboratory of Transcriptional Networks, Centre for Integrative Biology (CIBIO), University of Trento, TN, 38060 Italy, ^2^Molecular Mutagenesis and DNA Repair Unit, IRCSS Azienda Ospedaliera Universitaria San Martino-IST-Istituto Nazionale per la Ricerca sul Cancro, Genoa 16132, Italy, ^3^Department of Chemistry and Biochemistry, University of California San Diego, La Jolla, CA, 92093, USA and ^4^Chromosome Stability Group, Laboratory of Molecular Genetics, National Institute of Environmental Health Sciences, NIEHS, NIH, RTP, NC, 27709, USA

## Abstract

Structural and biochemical studies have demonstrated that p73, p63 and p53 recognize DNA with identical amino acids and similar binding affinity. Here, measuring transactivation activity for a large number of response elements (REs) in yeast and human cell lines, we show that p53 family proteins also have overlapping transactivation profiles. We identified mutations at conserved amino acids of loops L1 and L3 in the DNA-binding domain that tune the transactivation potential nearly equally in p73, p63 and p53. For example, the mutant S139F in p73 has higher transactivation potential towards selected REs, enhanced DNA-binding cooperativity *in vitro* and a flexible loop L1 as seen in the crystal structure of the protein–DNA complex. By studying, how variations in the RE sequence affect transactivation specificity, we discovered a RE-transactivation code that predicts enhanced transactivation; this correlation is stronger for promoters of genes associated with apoptosis.

## INTRODUCTION

The p53 family of transcription factors is composed of the proteins p53, p63 and p73 (1–3) that share an N-terminal transactivation domain (TA), a central sequence-specific DNA-binding domain (DBD) and an oligomerization domain (OD) within the C-terminal domain. An additional C-terminal sterile-α-motif, involved in protein–protein interactions, is present only in p63 and p73 proteins. Multiple isoforms are generated from alternative promoter usage and alternative splicing of the three genes, act as tetramers and influence many cellular pathways including cell proliferation, apoptosis, DNA repair, angiogenesis, metabolism and differentiation [(4,5) and references therein].

Biochemical assays point to comparable DNA-binding specificities for p53, p63 and p73, even though quantitative differences for certain DNA sequences have been also reported (6,7). Studies of genome-wide occupancy and gene expression reveal partially overlapping gene networks (8), but also many examples of genes exclusively targeted by p53, p63 or p73 (9–12). Several factors have been invoked to explain differences between *in vitro* binding and *in vivo* occupancy and expression (6). Such factors include differences in the protein–protein interactions for the less conserved N- and C-terminal domains of the p63 and p73 proteins, as well as variations in the chromatin landscape at target promoters (13).

Despite gene structure and protein function similarities, the overlap in cellular functions between p73, p63 and p53 is limited. For example, p53 knockout mice are viable and largely normal in embryonic development, but they die at an early age due to spontaneous cancers (14). p73-null mice appear normal at birth but display neurological, pheromonal and inflammatory defects resulting in death within 2 months (15), whereas p63-null mice die at birth and exhibit growth abnormalities, such as defects in ectodermal-derived tissues, a lack of limbs and epidermis, as well as absence of mammary, lacrimal and salivary glands (16,17). The p53 family members also appear to have different functions in human biology. p53 is a well-established tumour suppressor and is one of the most frequently mutated proteins in sporadic cancers. Moreover, *TP53* germline mutations are associated with the development of the cancer-prone Li-Fraumeni and Li-Fraumeni-like syndromes (18). Conversely, p63 is critical for correct development of ectodermal-derived tissues, whereas p73 contributes to neural and immune systems functions (15,16). Cancer development is rarely associated with p73 and p63 mutations; no genetic disorder has been linked to p73 (19), whereas heterozygous mutations in the p63 gene underlie a subset of human ectodermal dysplasia syndromes, which recapitulate the mice knock-out phenotype (20).

The p53-family response element (RE) has a loose consensus which, based largely on *in vitro* binding studies, consists of two decameric ½-sites separated by a short spacer (n): RRRCWWGYYY-(n)-RRRCWWGYYY (R = purine; W = A/T; Y = pyrimidine; n = 0–13, although for the majority of validated REs have n < 3) (21–23). However, as most sites that have been validated by *in vivo* studies contain mismatches from the consensus, transactivation selectivity among the p53 family proteins may, at least in part, be coded in the DNA sequence of the target REs. For example, p63 was shown to preferentially activate sequences with a G in the 5th and/or 15th position (with n = 0) within the core of the RE (RRRC**G**WGYYY) (24). Specific mismatches at the 10th and 11th positions appeared to contribute as well (7). A slight preference for G at the fifth position in the RE was also confirmed for p73 (6).

The crystal structures of all the members of the p53 family show that the DNA-recognition residues are conserved across the family (25–31). Similar to p53, human p73 DBD was shown to self-assemble as a tetramer (dimer of dimers) on a full-site RE. Nonetheless, there are differences in the dimerization and tetramerization interfaces of the p73 and p63 DBD tetramers compared with p53 that might account for variations in relative DNA-binding affinities (26). In particular, only p53 dimers would be able to engage in salt bridge interactions among residues E180 from the L2 loop of one monomer with R181 from the other monomer (29,32). Additionally, the p63 DBD/DNA structure differs from p53 in L1 loop conformation and in the intra-dimers surface (33). In p73, the conformation of loop L1 changes depending of the sequence of the second and third nucleotides of the quarter-site RE (34).

Mutations mainly in the L1 loop of the p53 DBD exhibit striking changes in transactivation specificity (35–37). Based on those results, we have carried out a mutation-driven comparison of the transactivation specificity of p53, p63 and p73. To this aim, the p73 S139F and T141A (L1 loop), S260N (L3 loop) alleles and the p63 S150F, T152A, S271N alleles, corresponding to p53 S121F, T123A, S240N alleles, were analysed for their transactivation activity on a wide panel of p53 REs, using our well-established yeast-based assay (38), along with functional analyses in human cells. A high degree of overlap in transactivation potential and specificity for full-length p53, p63 and p73 proteins was established. Specific positions within the RE involved in establishing direct protein–DNA contacts were linked to transactivation selectivity, a result supported by DNA-binding studies and the crystal structure of p73 S139F DBD bound to DNA. Correlations between the RE sequence and transactivation levels suggest a built-in DNA code for promoter selectivity towards p53 apoptotic genes.

## MATERIALS AND METHODS

### Yeast reporter strains, media and expression vectors

**Figure 1.**
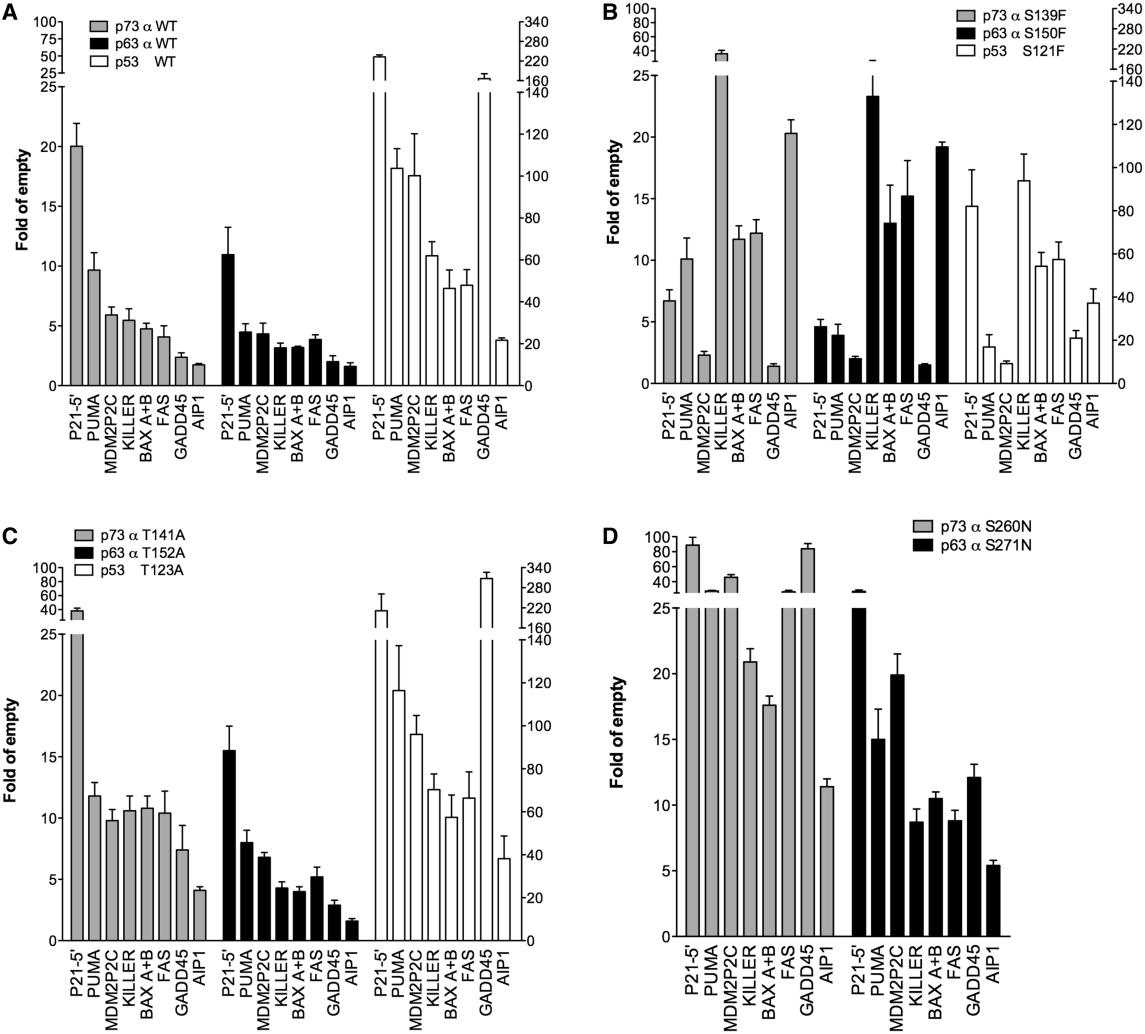
Evolutionarily conserved altered-function amino acid positions in p53 family proteins exhibit overlapping relative transactivation potentials. (**A**) Transactivation potential of WT p73, p63 and p53 expressed under the moderate constitutive *ADH1* promoter towards eight REs was determined using a yeast-based functional assay. (**B**) Transactivation potential of the p73 S139F and the corresponding p63 S150F and p53 S121F mutants. (**C**) Transactivation potential of the p73 T141A, the corresponding p63 T152A and p53 T123A mutants. (**D**) Transactivation potential of the p73 S260N, the corresponding p63 S271N. Presented in all graph is the fold-induction over the empty expression vector (pRS314) of mean luciferase activities normalized to unit of soluble proteins. Error bars plot the standard deviations of four biological replicates. The *Y*-axis on the left refers to the results with p53 alleles. The higher relative activity of p53 proteins is discussed in the text. The RE sequences are ordered based on decreasing WT p73 transactivation potential. For p73 and p63, TA alpha isoform proteins were examined.

**Figure 2.**
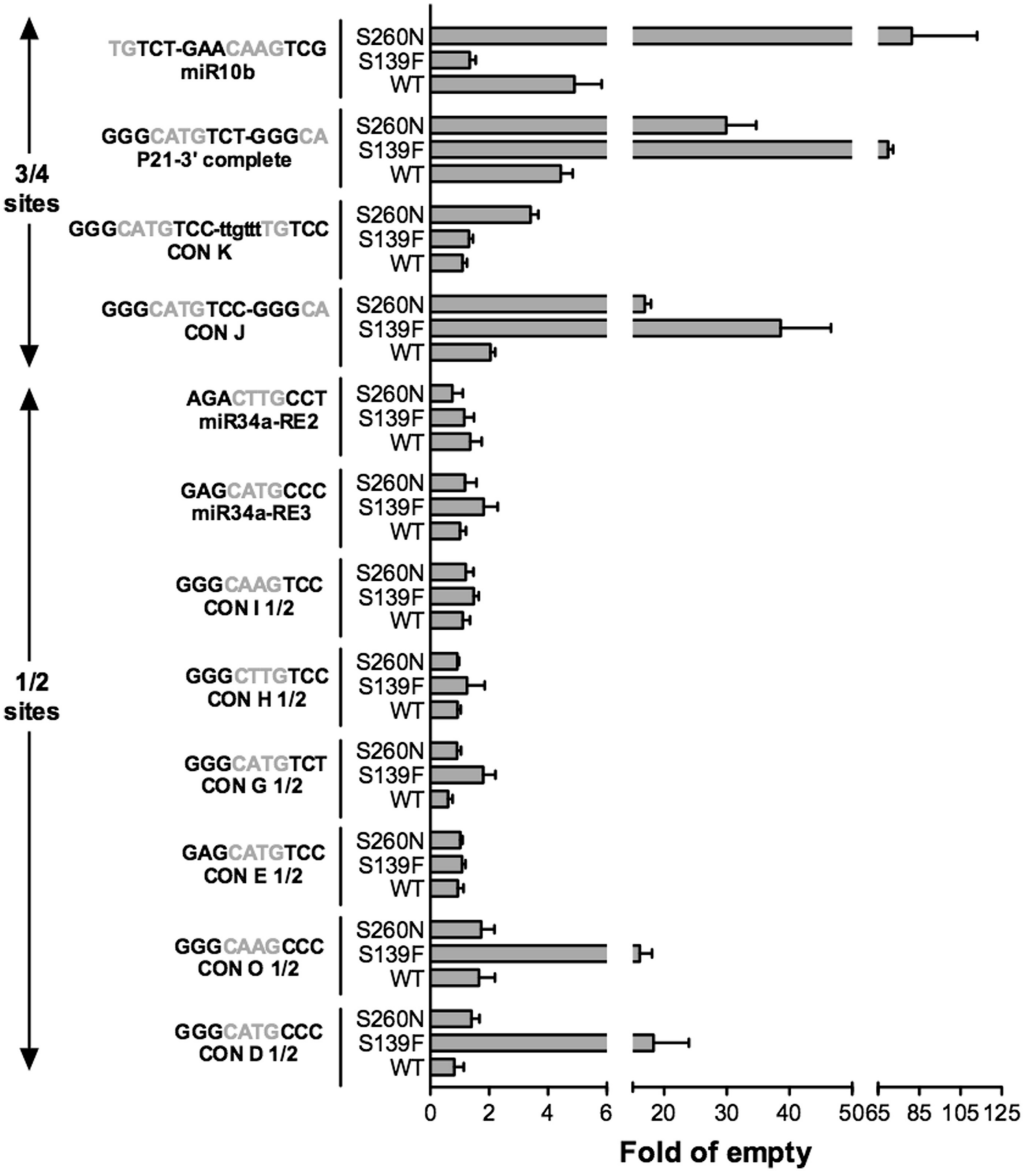
Altered function TA p73β mutants can activate transcription from non-canonical REs. Relative transactivation potentials of p73β alleles (WT, S139F and S260N) towards 12 non-canonical p53 REs comprising ¾- and ½-sites were determined using the yeast-based functional assay. The name and sequence of the 12 REs are shown. The core (GWWC) sequence is depicted in grey. Bars plot the mean fold of induction (normalized to OD_600_) over the empty expression vector (pRS314) and standard deviations of four biological replicates, computed as in Figure 1.

We used a panel of 53 *Saccharomyces cerevisiae* haploid reporter strains (yLFM-REs); all strains are isogenic except for the different p53 REs located upstream the luciferase reporter gene (39) (Figures 1, 2 and 5 and Supplementary Table S1). Thirty-three REs were obtained from promoters of human p53 responsive genes (Physiological REs, P-REs), whereas 19 were consensus REs designed ad-hoc (Synthetic REs, S-REs). A single p53-RE (m-fas) from a mouse ortholog was also analysed. All yeast strains were generated using the *Delitto Perfetto* approach (40). Yeast cells were grown in 1% yeast extract, 2% peptone, 2% dextrose with the addition of 200 mg/l adenine (YPDA medium). Plasmids for constitutive expression of wild-type (WT) p53, p73 and p63 proteins as well as the pRS314 empty vector were available (41–43). Yeast transformants were selected on minimal plates lacking tryptophan. For the construction of mutant alleles in the yeast expression vector, we adopted the experimental strategy previously described (44). A list of all the primers and restriction enzymes used are available on request.

### Luciferase assays in yeast

yLFM-REs transformants (obtained by LiAc method) were streaked onto minimal plates and processed as previously described (38,44). Luciferase activity, measured using a multilabel plate reader (Mithras LB940, Berthold Technologies, Bad Wildbad, Germany) following the manufacturer’s protocol (Bright Glo Luciferase Assay, Promega, Milan, Italy), was normalized either to units of soluble proteins (light unit/µg protein) or to optical density (OD_600_). For each reporter strain, the background luciferase activity was measured from pRS314-transformed clones and used to calculate the fold of reporter induction.

### Cell lines, culture conditions and expression vectors

The colon adenocarcinoma cell line HCT116 p53^−^^/^^−^ was obtained from B. Vogelstein (The Johns Hopkins Kimmel Cancer Center, Baltimore, Maryland, USA). Cells were maintained in RPMI medium supplemented with 10% Fetal Calf Serum (FCS) and antibiotics (100 units/ml penicillin, 100 µg/ml streptomycin) (Lonza, Milan, Italy).

The pcDNA3.1-p73 plasmids expressing the WT or mutant p73 alleles (*Cercopithecus aethiopicus*) were obtained from double digestion (XhoI/XbaI) of pTSG-p73β (43) and empty pcDNA3.1 vectors. The 5.4 kb fragment from pcDNA3.1 was ligated with a 3 kb fragment from pTSG-p73β containing also the 5′ Untranslated Region (UTR) of p73 gene. For the expression of human p53, we used the pC53-SN3 plasmid (45). The empty vector pCMV-NeoBam plasmid was used to keep constant the DNA amount to be transfected.

The pGL3-1138 (gift of Dr W. El-Deiry) and pGL3-P21-5′ reporter vectors that contain a 2.3 kb promoter fragment or the distal p53-RE of P21 gene (corresponding to the RE of yLFM-P21-5′ strain), respectively, were used in transient transfection experiments. The pGL3-1012, pGL3-MDM2 (gift of Dr M. Oren) and pGL3-AIP1 reporter vectors that contained fragments of the p53-responsive promoters from the BAX, MDM2 and AIP1 genes, respectively, were also tested (36). The pRL-SV40 plasmid, harbouring the luciferase gene from *Renilla reniformis* under the control of a constitutive promoter, was used to normalize with respect to transfection efficiency.

### Luciferase assays in mammalian cells

HCT116 p53^−^^/^^−^ cells were seeded in 24-well plates (6–8 × 10^4^ cells) and transfected using the *Trans*IT-LT1 transfection reagent (Mirus, Milan, Italy) according to the manufacturer’s instructions. The transfection mixture (500 ng/well) contained 250 ng of the different pGL3 promoter-derived p53 reporter plasmids, 200 ng of the expression or empty vector and 50 ng of the pRL-SV40 control plasmid. Cells were harvested 48 h after the transfection and luciferase assays were conducted as previously described (46).

### Real time PCR

HCT116 p53^−^^/^^−^ cells were harvested 24 h after transfection and washed with PBS before mRNA expression analyses. Total RNA was extracted using the RNeasy Kit (Qiagen, Milan, Italy) according to the manufacturer’s instructions. cDNA was generated from 2 µg of RNA by using the RevertAid M-MuLV First Strand cDNA Synthesis Kit (Fermentas, Milan, Italy). Real-time qPCR was performed on a RotorGene 6000 thermal cycler (Corbett Life Science, Ancona, Italy) using the KAPA Sybr green-based FAST qPCR MasterMix kit (Kapa Biosystems, Resnova, Rome, Italy). Primers used were selected using the Primer-BLAST online tool (http://www.ncbi.nlm.nih.gov/tools/primer-blast/) and sequences are available on request. Relative mRNA quantification was obtained using the ΔΔ_Ct_ method, where the Glyceraldehyde 3-phosphate dehydrogenase (GAPDH) and the β_2_Microglobulin genes served as internal controls.

### Western blot analysis

HCT116 p53^−^^/^^−^ cells were harvested 24 h after transfection and washed with PBS before soluble protein extraction. Cells were lysed in Radioimmunoprecipitation assay (RIPA) buffer supplemented with protease inhibitors (Roche, Milan, Italy), and soluble proteins were recovered and quantified using the BCA method (Pierce, Milan, Italy). In all, 50 µg of protein extracts was loaded on a 12% acrylamide gel and probed with specific antibodies: ER-15 (p73, Calbiochem, Merck, Darmstadt, Germany), DO-1 (p53, Santa Cruz Biotechnology, Heidelberg, Germany), C-19 (p21, Santa Cruz), SMP-14 (MDM2, Santa Cruz) and 6C5 (GAPDH, Santa Cruz). Immunoreactive bands were quantified using the ImageLab software (BioRad, Milan, Italy).

### Flow cytometry analysis

Early apoptotic response was visualized using the FITC Annexin V Apoptosis Detection kit (BD Biosciences, Milan, Italy) following the manufacturer’s recommendation. Briefly, HCT116 p53^−^^/^^−^ cells were seeded and transiently transfected with the empty or mutant expression vectors. Where appropriate, the p53 stabilizing agent 5-Fluorouracil (375 μM) was added to the p53-transfected cells as positive control. Twenty-four hours after transfection, cells were harvested, washed twice with cold PBS, before proceed with FITC Annexin V and Propidium Iodide (PI) detection. As controls unstained cells, cell stained only with FITC Annexin V (no PI) or cells stained only with PI (no FITC Annexin V) were used. Alternatively, for analysis of the cell cycle phases distribution the CycleTEST^TM^ PLUS DNA kit (BD Biosciences) was used. Cells were processed as previously described. Staining procedures were performed following the manufacturer’s recommendation.

#### Fluorescence polarization

The mutation S139F was introduced using the Quikchange site-directed mutagenesis protocol (Agilent Technologies, Santa Clara CA, USA) into the WT human p73DBD gene (residues 115–312) carried in the pET28 expression vector. Expression and purification were conducted as previously described (26). The binding affinity of the purified p73DBD S139F mutant for a RE sequence was determined by measuring the polarized fluorescence at different concentrations of protein and at a constant concentration of DNA. We used four DNAs: two 12 bp-containing, half-site REs (5′-tGGGCATGCCCa-3′ and 5′-cGAACATGTTCg-3′) and two 20 bp-containing, full-site REs (5′-GGGCATGCCCGGGCATGCCC-3′ and 5′-GAACATGTTCGAACATGTTC-3′). The fluorescein-labelled DNAs were dissolved in double-distilled water, annealed by heating to 95°C for 10 min and slowly cooled to room temperature. The protein solution (18 samples from 0 nM to 20 000 nM) was gently mixed with 50 nM of fluorescein-DNA, incubated on ice for 30 min and warmed up to room temperature before measuring fluorescence intensity using Hitachi F-2000 Fluorescence Spectrophotometer with excitation and emission wavelengths of 494 and 521 nm, respectively. The polarization was analysed to determine the fraction of DNA in complex with protein. The data were plotted with the log of concentration [log(nM)] on the *x*-axis and the percentage fraction bound on the *y*-axis. The plot was fitted to a sigmoidal dose-response curve to calculate the non-linear regression. The calculated EC_50_ was considered to be equivalent to the K_d_ of the protein–DNA complex, with the assumption, supported by analytical ultracentrifugation data, that one dimer was bound per 12 bp and one tetramer was bound per 20 bp.

#### Crystallization and structure determination

p73 proteins purification was conducted as described previously (26,34). The p73DBD S139F protein was screened for crystallization as a protein–DNA complex using the DNA sequences and conditions previously described for the WT p73DBD (26). The final crystals were obtained by mixing the protein at a 2:1 molar ratio with the annealed 20 bp oligonucleotide (5′-GAACATGTTCGAACATGTTC-3′). Using the hanging-drop method, 1 µl of the protein–DNA mixture was added to 1 µl of well solution. Diffracting crystals were formed in well solutions with 0.1 M Tris (pH 8.5), 50–125 mM sodium acetate and 20–24% (w/v) PEG 3350. Thin needle-like crystals formed after 1 day at room temperature, with slightly thicker crystals growing at lower sodium acetate and PEG 3350 concentrations. The crystals were washed in a cryo-protectant solution [0.1 M Tris (pH 8.5); 75 mM sodium acetate and 30% PEG 3350] and were frozen by plunging into liquid nitrogen. The 20 bp p73DBD S139F complex diffracted to 3.7 Å, and the diffraction data were collected in beamline 7.1 at the SSRL facilities. Indexing, integration and scaling of the diffraction data were done using HKL2000 (47). Crystal data and intensity statistics are given in Supplementary Table S2. The lattice system was determined to be primitive hexagonal and the space group P6_1_ with unit cell parameters a = b = 172.49 Å, c = 34.09 Å and γ = 120°. The asymmetric unit consists of two molecules of p73DBD S139F and one 10 bp half-site. The structure was solved by molecular replacement with the WT p73DBD dimer in complex with the 12 bp DNA as the initial model using the program Phaser (48). The resulting solution was refined using rigid body refinement, simulated annealing and energy minimization in Crystallography & NMR System (CNS) (49). The 10 bp half-site forms a continuous 20 bp DNA with the symmetry related molecule in the crystal. Omit maps were used for manual and real space refinement of the protein, DNA and water molecules in the program Coot 0.6.1 (50). Further refinement was carried out in CCP4 (51) using translation, libration and screw-rotation (TLS) refinement (52) with Non-crystallographic symmetry (NCS) constraints between the two protein chains to reduce the number of refined parameters. The structure was refined to *R*_work_/*R*_free_ = 30.3/31.6, and final model statistics are given in Supplementary Table S2. The PROCHECK program (53) was used to validate the structure, and 98.7% of the residues were in the most favoured and additionally allowed regions of the Ramachadran plot. Calculation of the DNA structural parameters and analysis was carried out using the program 3DNA (54).

## RESULTS

### Mutant analysis reveals conservation of L1 and L3 loop function within the p53 family

We have shown that mutations of a few evolutionary conserved amino acids in the p53 L1 and L3 loops, involved in contacting DNA (55,56), could lead to a complex change in the transactivation spectrum across many REs that also include changes in levels of transcription (39) (Supplementary Figure S1A and B). Similar changes in transactivation by corresponding mutant alleles in the p53 family members would support conservation of structure-function. We, therefore, compared transactivation potentials of p53 mutants S121F, T123A and S240N to the corresponding p73 mutants S139F, T141A, S260N and to the p63 mutants S150F, T152A, S271N, under the moderately expressing *ADH1* constitutive promoter.

WT p73, p63 proteins (α isoforms) and p53 had similar patterns of transcription activation across several REs with the exception of GADD45 RE, although induction was ∼10-fold higher with p53 (Figure 1A) (43,57).

The p73 S139F, the corresponding p63 S150F and p53 S121F alleles showed a ‘change-of-spectrum’ phenotype, namely, an enhanced activity on the KILLER, BAX A + B, FAS, AIP1 REs but a reduced activity on P21-5′, PUMA, MDM2-P2C, GADD45 REs (Figure 1B). The p73 T141A and S260N alleles along with the corresponding p63 T152A and S271N alleles were associated with increased transactivation for all p53 REs tested (Figure 1C and D). The increase, also referred to as super-transactivation (35), was the greatest for the p73 S260N allele, with GADD45 RE being activated more than 22-fold compared with WT p73 (Figure 1A and D). Overlapping results were obtained with WT p73 and p63 β isoforms (Supplementary Figure S2A and B). These results were highly comparable with data previously obtained for the corresponding p53 alleles, using an inducible expression system (39).

Previously, we found that the *ADH1*-based system for expression of p53 alleles could be used for all but p53 S240N, which inhibited yeast growth (35). As expected, detection of enhanced transactivation was limited for the p53 T123A allele under the *ADH1* due to the high level of p53-dependent reporter induction that approaches the technical limit of luciferase detection, but the change-of-spectrum phenotype by the p53 S121F allele was clearly observed (Figure 1B).

Overall, these results demonstrated an allele-dependent alteration in transactivation specificity shared by all the p53 family members. This suggests that the corresponding amino acid changes in the L1 and L3 loop have similar functions.

### The transcriptional impact of the p73 S139F and S260N alleles includes non-canonical REs

We extended our analysis of allelic changes to additional REs, focusing from hereon with the p73β isoform (WT, and two mutants: p73 S139F and S260N) because it showed the widest difference between WT and mutant alleles. Results for 48 REs (summarized in Supplementary Table S1) confirmed either the change-of-spectrum or the super-trans activity, respectively, for the two mutants. Non-canonical p53 binding sites, i.e. ½-sites and ¾-sites (43), were also examined (Supplementary Table S1 and Figure 2). The results demonstrated that, besides p53, WT p73 can also transactivate from ¾ sites. Unlike p53, it does not function from ½ sites. The functional features of p73 S139F and p73 S260N alleles were still evident using non-canonical REs. The change-of-spectrum mutant p73 S139F could even transactivate from a few ½-sites.

### p73 S139F and S260N generate altered-function and super-transactivation phenotypes when expressed in human cells

**Figure 3.**
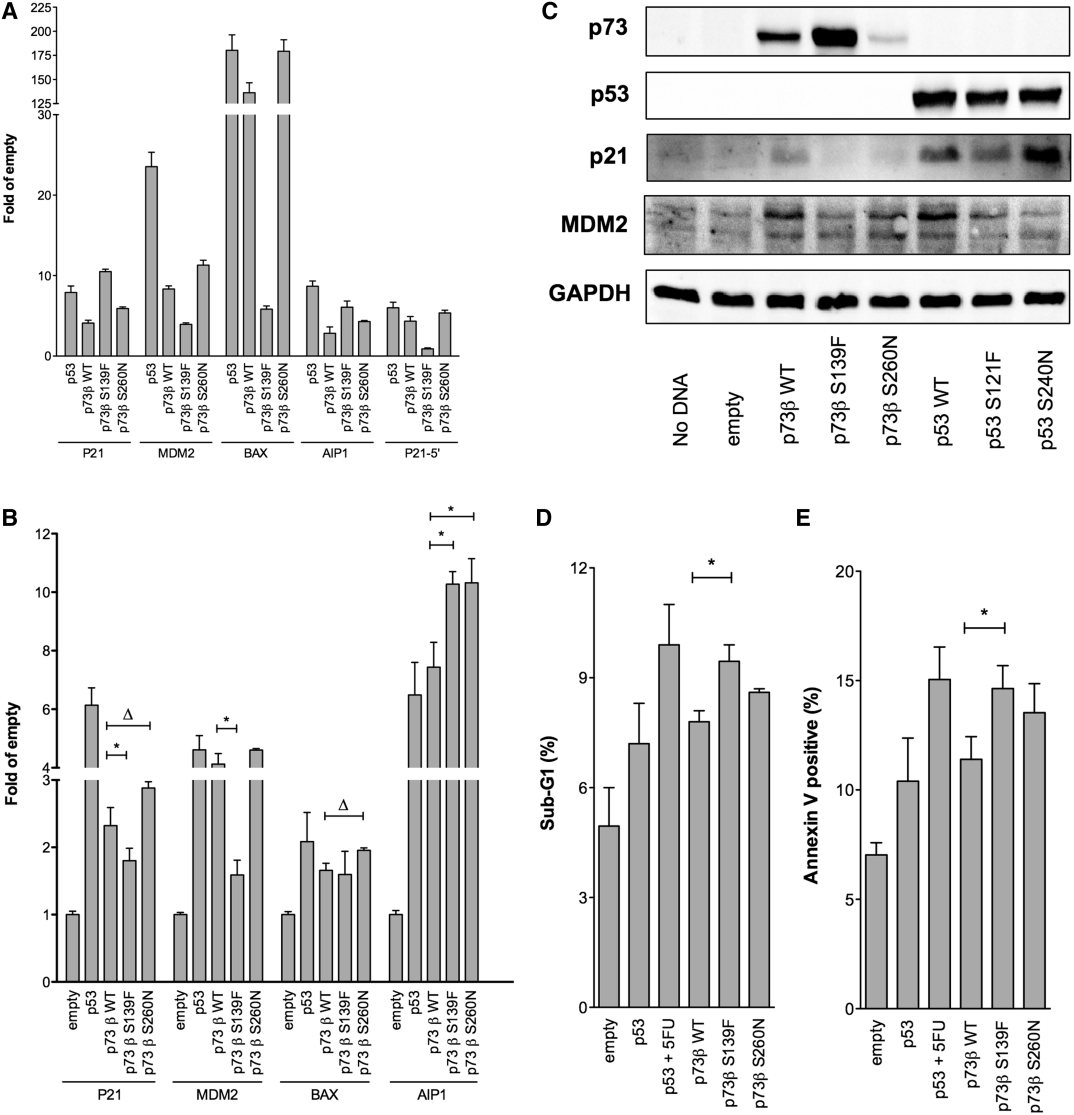
Altered transactivation and enhanced induction of apoptotic markers in p53 null HCT116 cells, on ectopic expression of p73 S139F or S260N. HCT116 p53^−/−^ were transiently transfected with expression vectors for p53, p73β alleles or an empty pCMV Neo-Bam control vector. (**A**) Gene reporter assays were performed by co-transfecting luciferase reporter vectors containing portions of the promoter sequence of the indicated p53-target genes along with the pRL-SV40 vector as a control for transfection efficiency. Presented are mean relative inductions over the empty expression vector and the standard deviations of at least three biological replicates. (**B**) Endogenous P21, MDM2, BAX and AIP1 transcript measurements were obtained by RT-qPCR 24 h post-transfection with the indicated p53 family expression vectors. Bars represent mean fold of induction normalized to the reference genes β2-microglobulin and GAPDH; standard deviations of at least three biological repeats. Asterisk indicates that the results of a two-tailed equal variance *t*-test is at least *P* < 0.01 (from left to right *P* = 0.008, *P* = 0.0005, *P* = 0.009 and *P* = 0.008, respectively). For Δ, *P*-value is at least <0.05 (from left to right *P* = 0.031 and *P* = 0.047). (**C**) Ectopically expressed p73/p53 alleles as well as endogenous p21 and MDM2 protein levels were measured by western blot 24 h post-transfection. GAPDH was used as loading control. (**D** and **E**) Proportion of Sub-G1 or Annexin V positive cells. Presented are the means and the standard deviations of at least three biological replicates. 5-Fluorouracil treatment 8 h after transfection with the p53 expression vector was included as a positive control (*P = 0.009).

To examine whether the p73β S139F and S260N alleles could lead, respectively, to the change-of-spectrum and super-trans phenotypes in human cells, they were transiently expressed in HCT116 p53^−^^/^^−^ cells along with a panel of luciferase reporter vectors. The S260N allele showed a modestly enhanced activity compared with WT p73 towards most of the reporters and BAX in particular (Figure 3A). The S139F allele showed higher activity towards AIP1 but reduced activity with the MDM2 intronic promoter and the P21-5′ RE, as predicted from the yeast-based assays. Interestingly, S139F was also super-transactivating with the P21 proximal promoter region (containing both P21-3′ and P21-5′ REs), suggesting that, with the plasmid-based assay, the proximal P21-3′ RE (super-transactivated in yeast) has a stronger impact on the reporter expression (Supplementary Table S1). The responsiveness of the BAX reporter, containing a 400 bp region of intron 1 of the gene, was markedly reduced with S139F. This latter result could not be directly compared with the yeast assays because the BAX RE tested in yeast contains an artificial duplication of one of the ½-sites of the RE in the BAX promoter.

The findings with the luciferase reporter assay led us to investigate the effect of the p73β alleles on endogenous gene expression by RT-qPCR (Figure 3B). The responses were similar to those obtained with the gene reporter assays and agreed with the yeast-based assays for MDM2 and AIP1 (Figure 3B). Interestingly, S139F was slightly less active than WT p73 towards the p21, possibly suggesting that, *in vivo*, the overall activity of the promoter is influenced by both distal (P21-5′) and proximal (P21-3′) REs that have opposite transactivation potential in yeast (see Figures 1 and 2). Also for BAX, there was a difference between luciferase and endogenous gene expression in HCT116 cells, with a WT-like phenotype for S139F in the latter endpoint, possibly suggesting that other sequences, previously reported in the BAX promoter (58) could be involved in the expression of the endogenous gene. The p73 S260N allele was equally or more active than WT p73 with all genes tested.

The relative expression both of the p73β and p53 alleles was examined on ectopic expression in HCT116 p53^−^^/^^−^ cells by western blot analysis (Figure 3C). p73 S139F appeared to be more abundant, whereas the p73 S260N protein levels were reduced compared with the WT protein. Instead, the three corresponding p53 alleles were equally expressed. We also measured the p73/p53-dependent induction of p21 and MDM2, which resulted to be reduced with the S139F/S121F relative to the WT proteins. The lower relative expression of p73-S260N limited the possibility to establish its higher potential to activate p21 and MDM2. However, normalizing for p73 proteins, the induction of both p21 and MDM2 by p73-S260N would be higher compared with WT p73. p53 S240N allele resulted in higher induction of p21 but slightly lower induction of MDM2. Although the relative differences in p21 and MDM2 expression could be in part dependent on the experimental setup, the results support the altered-function and super-transactivation features of the mutant alleles under study. The S139F/S121F alleles showed reduced transactivation capability towards the P21 and MDM2 p53-REs (Figure 1) and promoters (Figure 3A and B).

Given the effects on endogenous expression, we asked whether altered transactivation specificity might lead to a change in phenotypic outcome. Specifically, we monitored apoptotic endpoints in HCT116 p53^−^^/^^−^ cells ectopically expressing p73 S139F or S260N and compared with the ectopic expression of p53 with or without treatment with 5-fluorouracil. There was a significant increase (*P* < 0.05) in apoptosis induction for both mutant alleles compared with the WT p73 allele, based on both subG1 (Figure 3D) and annexinV staining (Figure 3E).

### The p73 S139F change-of-spectrum phenotype is determined by direct read-out of purines and pyrimidines and by the CWWG core RE sequence

To determine whether the p73 S139F phenotype could be associated with specific sequence features of REs, we observed that, compared with the WT protein, p73 S139F exhibited enhanced transactivation with 22 REs (S139F/WT > 1.5) (blue highlight in Supplementary Table S1), reduced transactivation with 9 REs (S139F/WT < 0.67; orange highlight in Supplementary Table S1) and similar activity with 6 REs (0.67 > S139F/WT > 1.5) among the 37 full-site REs that were previously tested.

**Figure 4.**
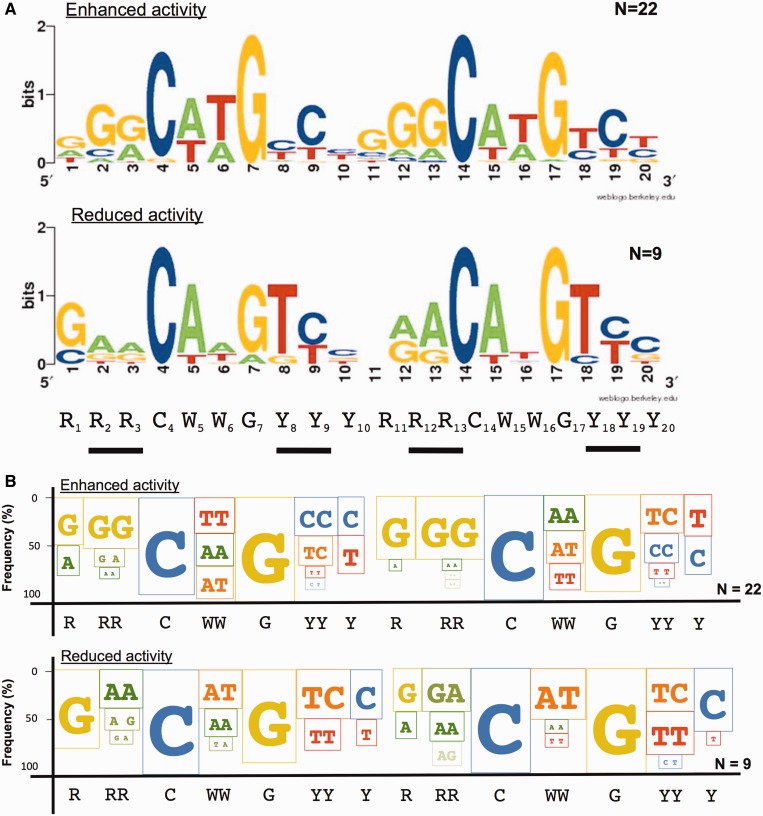
Dinucleotide signatures associated with enhanced or reduced transactivation potential by p73 S139F.p53 REs that were active with WT p73 were grouped according to enhanced (*n* = 22), reduced (*n* = 9) or nearly equal (*n* = 6) transactivation potential with p73 S139F (see Supplementary Table S1 for details on the cut-off of the WT/S139F ratios for the three classes). Dinucleotide sequences in the purine, pyrimidine and WW positions flanking the conserved C and G appeared to be correlated with the altered transactivation specificity. Therefore, instead of a conventional web-logo summary (**A**) (WebLogo 3, http://weblogo.berkeley.edu/logo.cgi), we developed a graphical summary that takes into account frequency of occurrences for paired nucleotide sequences (**B**). Plotted in this new version of the logo, where size is proportional to frequency, are only consensus changes. This graphical summary highlights how enhanced transactivation is associated with a lower frequency of CATG core sequence and with the GG, in RRC, and CC, in GYY, sequences. The p53 consensus sequence is indicated. As for the traditional logo view, spacers between half sites are omitted in the graphical summary.

DNA sequences for the two groups of REs showing opposite relative transactivation potentials were examined for distinctive features, using the WebLogo tool for a graphical summary. The group of 22 REs appeared to be characterized by an over-representation of Gs at position 2 and 3 (and 12 and 13) and Cs at position 8 and 9 (Figure 4A).

As the WebLogo tool only calculates the nucleotide frequencies for each position independently, without considering pairwise interactions or correlations between adjacent nucleotides, we expanded the WebLogo view analysis to highlight the frequency of purine or pyrimidine dinucleotides flanking the CWWG core as well as the frequency of WW motifs within the core (Figure 4B). For example, the WW motif has been found to be critical for transactivation with AT exhibiting the highest and TA the lowest activity, a finding that was recently related to differences in DNA torsional flexibility (59–61). A distinctive *RR = GG* and/or *YY = CC* signature (RR: G_2_G_3_ or G_12_G_13_ and/or YY = C_8_C_9_ and C_18_C_19_-herein referred to as *RY* signature) was also evident for the group of 22 REs that are super-transactivated by p73 S139F.

p73 S139F showed enhanced transactivation for REs containing at least one RY signature. The enhanced activity was proportional to the number of RY signatures (Supplementary Table S1). Conversely, reduced transactivation compared with WT p73 was associated with the lack of the RY signature (i.e. 0/4 RY). Differences at the level of the two WW motifs in the core sequence of the REs were also noted: TT or AA was more frequent in the group of the 22 super-transactivated REs (28/44 versus 5/18, *P* = 0.013, Fisher’s exact test).

With regard to p73 S260N allele, the super-trans phenotype was clear for most of the sequences tested (39/50 p53 REs, 78%), with the frequency increasing up to 89% (34/38) when only the canonical full-sites were considered, suggesting that this transactivation phenotype associated with allele is independent of the signatures identified for p73 S139F, potentially related to increased thermodynamic stability (62).

### *Ad hoc* permutations of the consensus RE confirm the sequence-dependent specificity of p73 S139F

**Figure 5.**
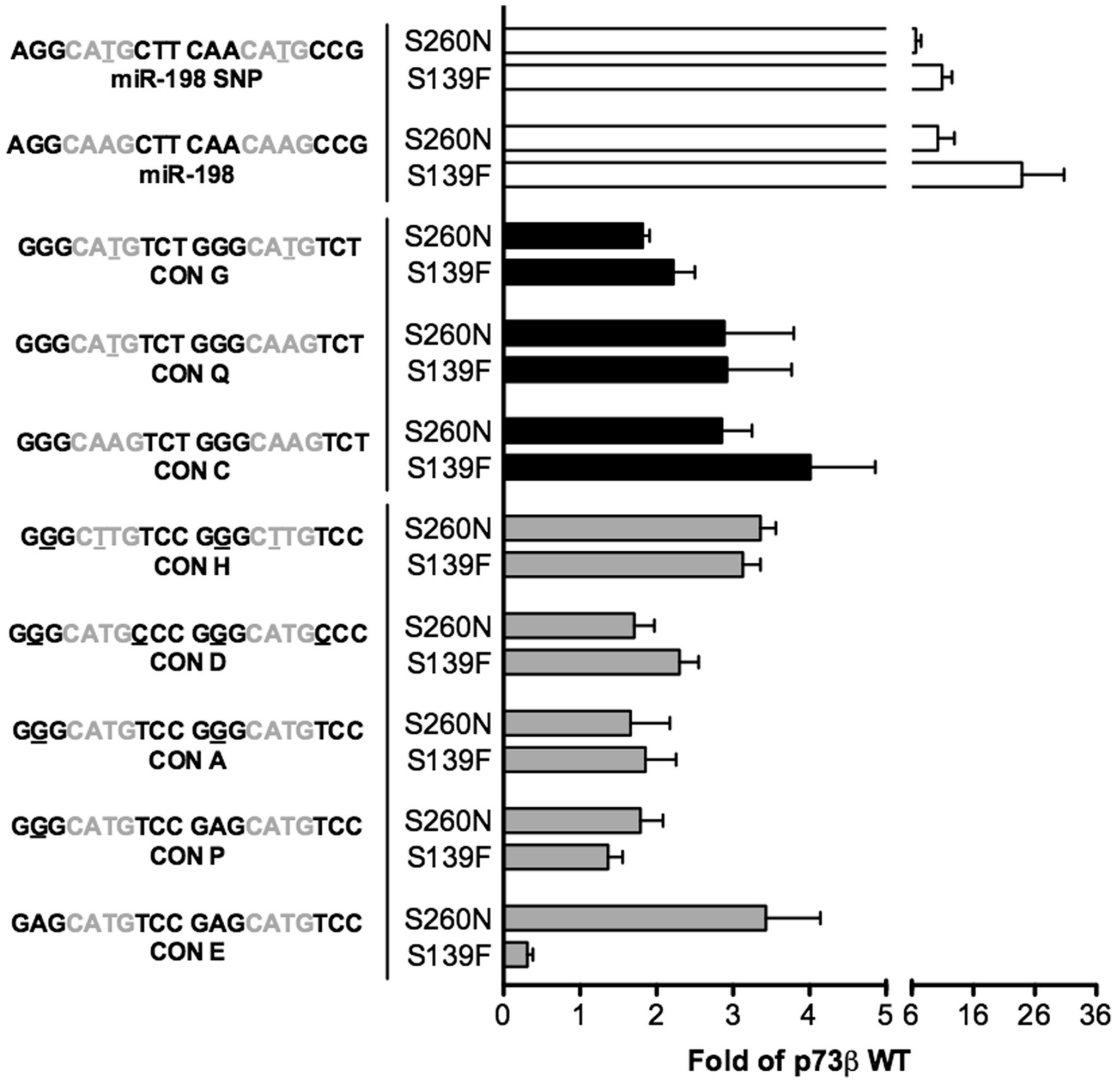
Ad-hoc permutations of p53 REs confirms a role of specific sequence features in mediating altered transactivation specificity by p73 S139F. Starting from CON E, CON C or miR-198 REs, 1 or 2 nt changes (underlined) were introduced in the core (CWWG, depicted in grey) or the core-flanking R and Y positions. The relative transactivation compared with WT p73 was measured for p73 S139F and S260N. Presented are the means and standard deviations of four biological replicates of the luciferase activity (normalized to OD_600_) measured in the various yeast strains. Results are expressed as transactivation ratio of p73 S139F or S260N over WT p73β. The colours of the bars group the permutated REs to be compared.

To investigate further the effect of the RY signatures and the core sequences in transactivating phenotypes, specific changes were created starting from a few consensus p53 REs (Figure 5). p73 S139F showed reduced transactivation with the ConE RE, consisting of two repeats of G*AG***CATG***TC*C sequences, having no RY among the four possible sites, and ATs as WW signature. We substituted the A at position 2 with a G, generating an RE with 1 of 4 RY signatures (ConP). This single change resulted in increased transactivation by p73 S139F compared with the WT p73 allele (Figure 5). Additional changes resulting in 2/4 RY RE (ConA) or a 4/4 RY RE (ConD) led to a further, modest increase in p73 S139F relative transactivation that was greatly enhanced when both WW sequences were converted from AT to TT (ConH).

The effect of the WW feature was also evaluated starting from 2/4 RY REs (ConC and miR-198). The enhanced relative transactivation by p73 S139F was reduced when changes were introduced resulting in the AT motif in either one (ConQ, miR-198 SNP) or both core sites (ConG) (Figure 5). p73 S260N continued to show the super-trans phenotype with all these REs. However, the extent of enhanced transactivation was in part affected by the level of responsiveness of the REs (e.g. compare CON E and CON A in Supplementary Table S1 and Figure 5).

### The p73 DBD S139F mutant binds DNA cooperatively

**Figure 6.**
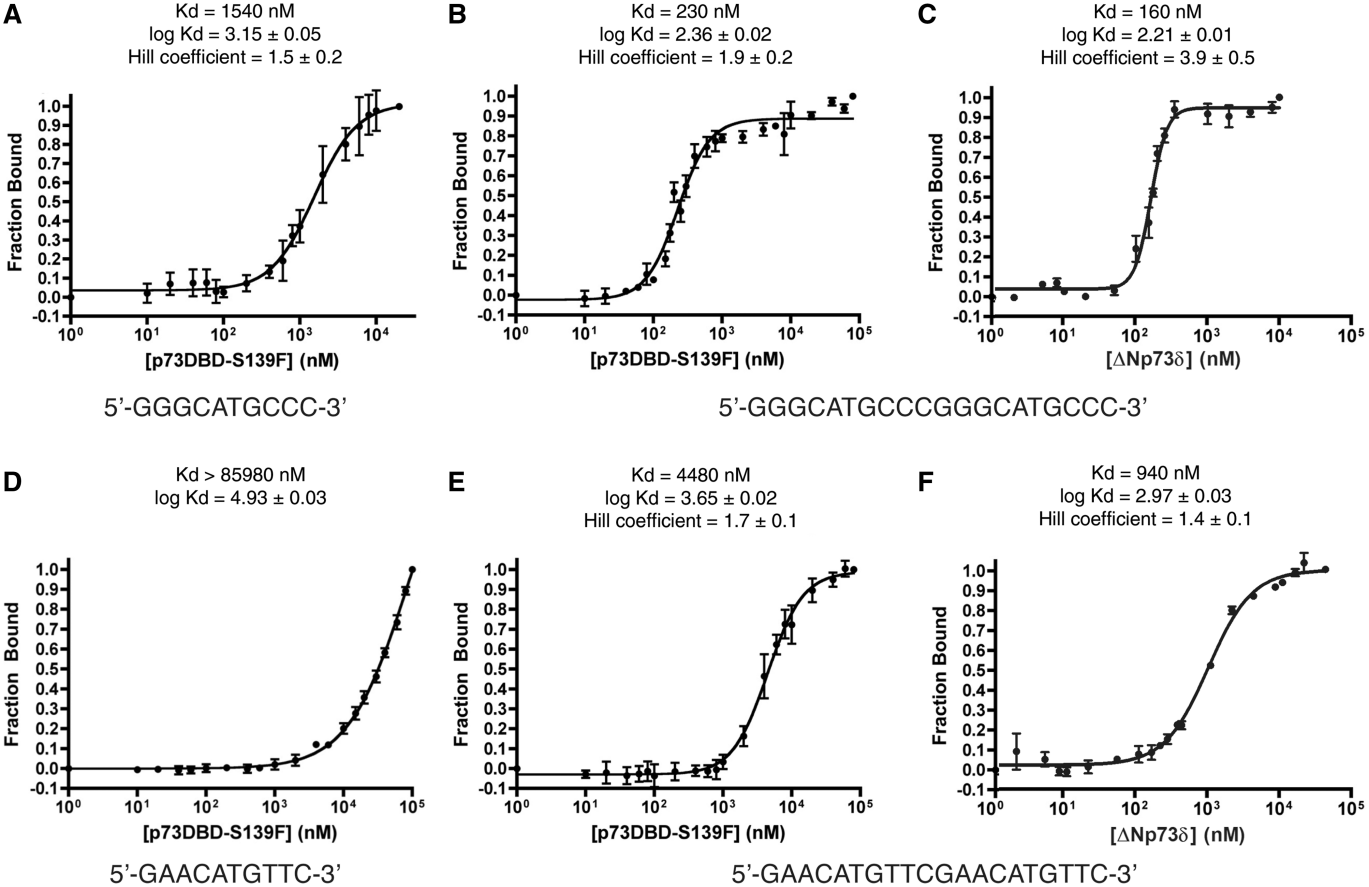
DNA-binding constants of the p73 S139F DBD mutant and the ΔNp73δ isoform for GGGCA and GAACA sequences**.** (**A**) The plot shows the fraction of fluorescein-conjugated GGGCA ½-site RE bound versus the added concentration of pure p73DBD S139F mutant. The sigmoidal dose-response curve was fit with a Hill coefficient different than 1. The error bars indicate the standard deviation between three data sets. The K_d_ was the concentration of p73 S139F DBD where 50% of the DNA substrate was bound to the protein. (**B**) The same procedure was repeated with the GGGCA full-site RE, showing even tighter binding with a K_d_ ∼7-fold lower in value. (**C**) Same as (B), but for the ΔNp73δ isoform. (**D**) Same as (A), but with a GAACA ½-site RE. (**E**) Same as (B), but with a GAACA full-site RE. The GAACA REs have much lower binding affinity than their GGGCA counterparts. (**F**) Same as (E), but for the ΔNp73δ isoform. The p73 S139F mutant has similar DNA affinity and specificity that the ΔNp73δ isoform.

The correlation between relative transactivation potential and DNA-binding affinity of p73 S139F was then investigated using purified mutant DBD protein with four DNA consensus sequences and measuring fluorescence polarization. The dissociation constants (K_d_) were determined to be 1540 nM for the 12mer GGGCA ½-site (tGGGCATGCCCa) and 230 nM for the 20-mer GGGCA full-site (GGGCATGCCCGGGCATGCCC) (4/4 RY signature) with Hill coefficients of 1.5 ± 0.2 and 1.9 ± 0.2, respectively (Figure 6A and B). The K_d_’s of the 12mer GAACA ½-site (cGAACATGTTCg) and the 20mer GAACA full-site (GAACATGTTCGAACATGTTC) (0/4 RY signature) were determined to be >85 980 nM and 4480 nM with a Hill coefficient of 1.7 ± 0.1 (Figure 6D and E). Because the difference in DNA affinity of the mutant S139F towards the GGGCA full-site was 10 times stronger (230 nM) than what we had observed for the p73 DBD WT (2387 nM) (26), we decided to further explore the nature of this difference with proteins that contained the OD. We measured the binding affinity of the WT ΔNp73δ isoform for both 20mer sequences obtaining K_d_ values of 160 and 940 nM with the GGGCA and GAACA sequences, respectively (Figure 6C and F). Interestingly, we observed that the S139F mutant behaves in a similar fashion as the ΔNp73δ isoform that includes both the DNA-binding and the ODs. Both proteins show cooperativity, and both have the ability to preferentially bind to the GGGCA 20mer. Thus, S139F binds cooperatively to both full-site sequences, whereas the different K_d_ values were consistent with its transactivation properties according to the RY signature.

#### Phenylalanine 139 distorts the structure of the L1 loop

**Figure 7.**
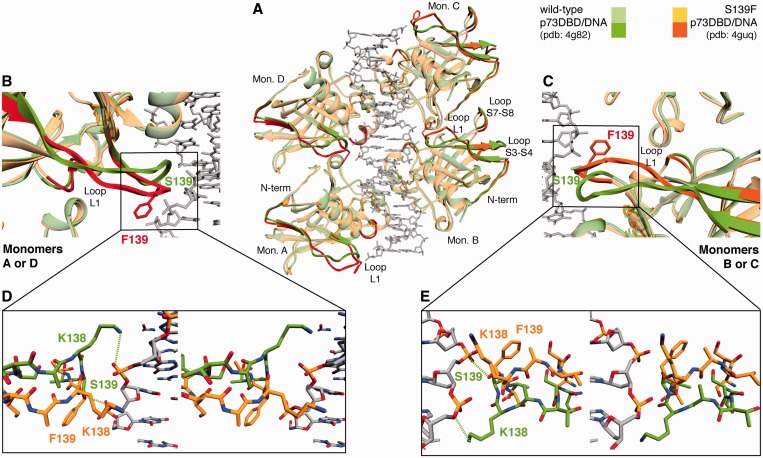
Crystal structure of the p73 S139F DBD mutant in complex with a full-site RE and in comparison with the WT p73 DBD structure. (**A**) The p73 S139F DBD tetramer (yellow–orange) in complex with a 20mer GAACA DNA (grey) superimposed to the p73 WT tetramer (green–dark green) confirms the C2 molecular symmetry observed in all the DBDs of the p53 family. The regions with a root means square difference greater than 1.5 are in darker colours (orange for the mutant F139 and dark green for the WT). Besides changes in loop L1 and the N-terminus of all monomers, monomers B and C show differences in loops S3-S4 and S7-S8 that are far from the DNA-binding surface. (**B** and **C**) Ribbon display of the comparison of the secondary structure of loop L1 between the p73 S139F DBD–DNA complex structure (orange) and the WT p73 DBD-DNA complex structure (dark green) for both monomers A or D (Figure 7B) and monomers B or C (Figure 7C). In both unique monomers, loop L1 moves away from the DNA. (**D** and **E**) Stereo view of the atomic display of the comparison of the secondary structure of loop L1 between the S139F p73 DBD–DNA complex structure (orange) and the WT p73 DBD–DNA complex structure (dark green) for both monomers A or D (Figure 7D) and monomers B or C (Figure 7E). In the WT p73DBD, S139 is at hydrogen bond distance (3.4 Å) of an oxygen atom in the DNA phosphate backbone. Instead, in the p73 S139F mutant DBD, the aromatic ring of the phenylalanine is rotated away from the DNA backbone and does not contact the DNA. Moreover, although in WT p73 the terminal amine of K138 is 3.8 Å from the oxygen in the phosphate backbone, in the S139F mutant, K138 is significantly displaced and cannot form the same interactions as in the WT. The p73 WT DBD–20mer complex structure is deposited in the PDB with ID: 4g82 (34), and the p73 S139F DBD–20mer complex structure is deposited in the PDB with ID: 4guq.

The crystal structure of p73DBD S139F in complex with the 20mer GAACA full-site (0/4 RY signature) was determined to 3.7 Å resolution (PDB ID: 4guq). The configuration of the protein–DNA tetramer complex is consistent with the C2 molecular symmetry found in the structures of the WT DBDs of the three members of the p53 family (Figure 7A) (25–31,33,63,64). As observed before for five structures of WT p73 DBD bound to different DNAs (26), each monomer recognizes a ¼-site RE. In the rest of the description, we compare the structure of the S139F mutant with the structure of the WT bound to an identical 20mer GAACA full-site (PDB ID: 4g82) because both crystals have the same space group, unit cell dimensions and crystal packing (34). Moreover, the asymmetric unit in the crystals for both proteins, the WT and the S139F mutant, has a dimer with half-site RE. As a result, in the final structure, both dimers are identical: dimer AB is the same that dimer DC (monomer A = monomer D and monomer B = monomer C) (Figure 7A). At the same time, the one-half RE DNA bound to the dimer AB has identical structure to the one bound to dimer DC.

As mentioned, the overall structure of the WT and S139F mutant is alike with a root mean square deviation (r.m.s. dev.) between the α carbons of the WT p73 DBD and the S139F mutant of only 1.15 (Figure 7A). As it could be expected, the larger structural differences reside in L1 that carries the mutation S139F. Few other regions have an r.m.s. dev. larger than 1.5 (highlighted in darker colours in Figure 7A). For example, monomers A (or D) of WT and S139F mutant also differ in the small amino 204–207 acid stretch of loop L2B (1.6 r.m.s. dev.) that participates in the B-D tetramerization interaction of the tetramer in complex with a 2 bp spacer (26). For the other monomers, B (or C), of WT and mutant, they differ in three other regions besides loop L1: amino acids 161–172 in loop S3-S4 (1.5 r.m.s. dev.), amino acids 206–208 again in loop L2B (1.6 r.m.s. dev.) and amino acids 245–249 in loop S7–S8 (1.5 r.m.s. dev.). The changes seen in the monomer B of mutant S139F in regions other than loop L1 appear to be in response to the N-terminus movement that occurs as a consequence of the rearrangement of loop L1 that seems to pull the N-terminus closer to the DNA and change the interactions of the loops S3-S4 and S7-S8 that pack against the N-terminus. The two loops between strands S3-S4 and S7-S8 are located in the opposite face of the DNA region, and they move towards the adjacent monomer. The mutation S139F may be affecting the packing of loops S3-S4 and S7-S8 by moving strand S1 that packs against strand S3. As a result of loop S7-S8 movement towards the adjacent monomer, the total buried tetramerization interface increases from 979 Å^2^ in the WT tetramer to 1188 Å^2^ for the mutant S139F tetramer. Such enlarged tetramerization interface could explain the observed cooperativity.

Nonetheless, the larger structural differences between the WT and the S139F mutant are in loop L1. Loop L1 carries the mutation S139F that is in close proximity to the first purine nucleotide of the ½-site RE (Figure 7B and C). However, loop L1 in the S139F mutant is moved further away from the base pairs in comparison with loop L1 in WT p73 DBD (26). The side-chain of Phe139 makes hydrophobic intra-loop contacts with the C-beta of Ser135 and Ala137 (Figure 7D and E). In one monomer of each dimer in the WT p73 DBD structure (PDB ID: 4g82), the amine N of Ser139 forms a hydrogen-bond with the DNA backbone phosphate (Figure 7D and E). In both monomers A (D) and B (C), the Phe139 residue in the p73 DBD S139F mutant is rotated away from the DNA molecule owing to the hydrophobic repulsion between the charged phosphate in the DNA backbone and the aromatic phenylalanine ring (Figure 7D and E). The S139F mutation changes the conformation of loop L1 by removing the hydrogen-bonds from K138 and S139 that contact DNA in the WT protein (26). Many residues in the N-terminus and loop L1 have B-factors higher than 100 Å^2^ [residues 133–140 in monomer A (D) and residues 121–140 in monomer B (C)]. Such high B-factor values indicate that these regions of the protein are highly flexible. In Supplementary Figure S5, we show a representative area of the 2Fo-Fc map that we used to model these regions, particularly loop L1. An unbiased omit map showed the backbone density, but it was more discontinuous and difficult to use for model building, strengthening the conclusion that these regions of the S139F mutant have no unique conformation in the crystal. Lys138 does not contact the DNA in either monomer, but, owing to the flexibility of loop L1, it cannot be discarded that Lys138 could transiently contact the DNA, but in a manner clearly different than the WT p73.

## DISCUSSION

### Conserved transactivation capacities among p53 family proteins

The p53 family of sequence-specific transcription factors can modulate an impressive variety of biological functions that are carefully tailored to specific cellular stresses. Multiple signal transduction pathways regulate p53 protein activities via transcriptional, post-transcriptional, translational and post-translational mechanisms (4,13,65,66). Different genetic approaches in mouse models or derived embryonic fibroblasts have consistently demonstrated that each p53 family member is associated with a distinct major function (20,67,68). However, all three genes participate in critical aspects of the DNA damage response pathways, including the induction of apoptosis and cell cycle arrest. In addition, all three genes contribute to maintaining genome fidelity, with some degree of tissue specificity (69), and with direct cooperation in the modulation of common target genes (70).

Many experimental approaches indicate that the transactivation-proficient proteins produced by the three genes (p53 full-length and the TA isoforms of p63 and p73) exhibit strong similarities in their DNA-binding specificities (6,71). This is consistent with the high sequence similarity (∼60%) between the DBDs and the conservation of all residues that contact the DNA. At the same time, the regulated transcriptional networks only overlap partially (7,24,72,73). As the sequence conservation among family members is higher for the DBD region than for the N- and C-termini, the selective binding to protein partners, influenced by post-translational modifications (74), has been proposed as a contributor to transcriptional specificity (4). Possible differences in the quaternary structures of the p53 family proteins have been revealed by the structural comparison of the tetramerization domains within the p53 family (6), by the propensity to form heteroligomers among the members of the p53 family (75), and by the studies of the flexible interactions between N- and/or C-terminus with the DBD (76). Such studies revealed possible differences in the quaternary structures of the p53 family proteins beyond the subtle DNA-binding affinities differences seen for the isolated DBD. Along this, recent crystal structures of p53 family DBD domains with different DNA sequences support the view that there are p53-, p63-, p73- specific dimerization and tetramerization contacts within the DBD that can affect protein–DNA interactions (26,30,33), and, particularly in p73, the conformation of Lys138 changes depending on the sequence of the RE (34).

In this work, we compared p53, p63 and p73 function using a well-defined transactivation assay in a reconstituted yeast-based system that enables us to measure relative transactivation capacity of each protein towards different REs cloned at the same position of a reporter gene inserted in a specific chromosomal locus (38,57,60).

Compared with p53, p63 and p73 showed overlapping relative transactivation profiles based on eight REs (Figure 1A and B) both in terms of the extent of transcriptional upregulation and the weaker activity towards the GADD45 RE. These discrepancies can be, in part, related to higher steady-state p53 protein levels in yeast, as revealed by epitope tagging (HA) and western blot analysis (Supplementary Figure S3A). Reducing p53 protein levels using the inducible *GAL1,10* promoter system resulted in p53 transactivation potential becoming more like that of p63 and p73 (Supplementary Figure S4).

The low level of p63 and p73 protein expression in the yeast system could also explain the lack of activity of these proteins with non-canonical ½-site REs that are activated by WT p53 only at high levels of protein expression (43). It is important to point out that almost all the full-site REs tested in this study did not contain a spacer separating the ½-site REs (Supplementary Table S1). As noted earlier in the text, we have recently shown that p73 appeared to be much more sensitive than p53 to the impact of short spacers separating ½-site REs. This result supports the view that differences in the tetramerization interfaces between p53 and p73 represent a source of phenotypic diversity among p53 family members (26).

Besides comparing WT proteins, we examined the effect on p63 and p73 of single amino acid substitutions previously found by us and others to alter p53 transactivation specificity (35,77,78). The results reveal that either the enhanced transcriptional activation or the altered target gene specificity of the different mutants is highly reproducible (Figure 1A and B and Supplementary Figure S2), indicating a high level of functional conservation. In particular, p73 T141A and S260N and p63 T152A and S271N alleles behave as super-transactivating mutants, whereas p73 S139F and the p63 S150F alleles were change-of-spectrum mutants.

Further exploring a mutation that could lead to enhanced but also reduced transactivation depending on the RE sequence was appealing because it could allow the exclusion of experimental biases, like differences in protein stability, solubility or nuclear localization in yeast, as such a bias is expected to affect similarly the results with all REs. Western blot analysis revealed that the relative steady-state protein expression was lower for p73 S139F and particularly for S260N compared with WT p73, suggesting that the enhanced transactivation or super-trans phenotype would be underestimated (Supplementary Figure S3B). Moreover, the altered function phenotypes were confirmed at different levels of expression (Supplementary Figure S2C).

### An RE-transactivation code defines the altered transactivation selectivity by p73 S139F

By comparing the transactivation potential of the p73 S139F mutant with the one for the WT protein for 48 p53 RE sequences, we identified general RE features that enhance or reduce activity. Considering the consensus motif RRRCWWGYYY ½-site, we identified sequence motifs corresponding to the WW central sequence within the CWWG core, and to the immediately flanking RR-YY sequence (RY signature). We developed a logo display to highlight these features (Figure 4B). Reduced transactivation with respect to WT (transactivation ratio < 0.67) was associated with the absence of GG (positions 2, 3 and 12, 13) or CC (positions 8, 9 and 18, 19) dimers in the consensus sequence (RY = 0/4), whereas enhanced transactivation (transactivation ratio > 1.5) was associated with the GG presence and was proportional to the number of RY signatures (Figures 4 and 5). In addition, enhanced transactivation was coupled to the lack of the AT motif within the WW signature for at least one ½-site. Furthermore, there appears to be a position bias for these RY motifs within the full-site RE sequence (Supplementary Table S1). For example, S139F also exhibited enhanced transactivation towards seven REs containing one RY motif. In all seven cases, the RY motif was in the second or third monomer sequence (i.e. in between the two core sites). Hence, inter-dimer contacts in the tetramer assembly are hypothesized to play a role in the altered transactivation potential.

Recently, enrichment for Gs and Cs flanking the core RE was found to impact p53 DBD dissociation kinetics (61). Previous studies with functional assays, including ours in yeast, indicated that the CATG sequence is associated with higher transactivation potential of WT p53. This result was not only attributed to higher torsional flexibility of this sequence motif (59,79) but also to higher binding affinity and occupancy (43). Interestingly, the WW positions have no direct contacts with the protein in the p53 crystal structure (29). Overall, the p73 S139F and the corresponding p53 and p63 mutants exhibited a preference for relatively rigid RE sequences, with the RY motifs contributing higher kinetic stability, as expected for high cooperative binding (59,61).

Biochemical assays confirm the importance of the presence of the RY signatures for cooperativity. Our DNA-binding assays with two different RE motifs [GGGCA (i.e. 4 RY signature) and GAACA (i.e. 0 RY signatures)] using fluorescence polarization revealed that the p73 S139F mutation affects DNA-binding cooperativity. Such cooperativity was not previously observed for the WT p73 DBD with ½- or full-site Res, when all sequences had a K_d_ ∼2000 nM (26). This contrasts with the p73DBD S139F, which shows clear cooperativity and a broader specificity. The mutant binds the sequence GGGCA at least 20 times tighter than the GAACA sequence (Figure 6A–D). Similar effects have been seen for the p53 S121F in gel shift assays with purified protein (80) and in relative occupancy at natural promoter sites mutant (36,78). We also observed that the DNA specificity and cooperativity of the S139F DBD mutant is also seen for the WT ΔNp73δ isoform (Figure 6E–F). In conclusion, the isolated S139F mutant DBD exhibits a specificity and cooperativity that the WT DBD does not, and, through an unknown mechanism, the S139F mutant resembles similar specificity and cooperativity than isoforms that contain the DNA binding and ODs.

### Structural consequences of the p73 S139F amino acid change

As mentioned earlier in the text, the crystal structures of all the members of the p53 family show that the DNA-recognition residues are conserved across the family while the dimerization and tetramerization interfaces are different (25–31). Understanding the structural similarities and differences between the members of the p53 family will help us to describe in molecular terms how each protein promotes a different, but somewhat overlapping, gene expression pattern. Comparing structures of the WT p73 DBD bound to five different REs, we have observed that K138 senses the sequence of R_2_R_3_ positions preceding the core RE sequence (34). If both positions have guanine, the positively charged ε-amino group of K138 forms hydrogen bonds with the O_6_ of both guanines; if only adenines are present in both positions, K138 does not contact them; and, if there is only one guanine and one adenine, K138 from one monomer contacts the guanine, while the K138 in the other monomer does not contact the DNA bases (34).

The crystal structure of the p73 S139F DBD in complex with the GAACA full-site RE also shows that loop L1, where K138 and the mutation S139F are located, is important for RE recognition. Such differential DNA binding seems to lead to different levels of gene transactivation. There are two main structural consequences of mutating the residue 139 from serine to phenylalanine: first, loop L1 suffers a conformational change that breaks the hydrogen bond network that keeps K138 and S139 in contact to the DNA; second, the movement of the loops between the strands S3-S4 and S7-S8 increases the tetramerization surface.

Our main structural observation is that the mutation S139F increases the flexibility of loop L1, together with more subtle structural rearrangements in the N-terminus and the loops S3-S4 and S7-S8. The observation that p73 S139F DBD has a much higher K_d_ than the WT p73 for the GAACA ½-site RE correlates with the destabilization of loop L1 in the crystal structure of the mutant with the GAACA RE. The destabilization of loop L1 structure promoted by the S139F mutant is also likely to occur in the case of the GGGCA sequence. Loop L1 and K138 are in contact with DNA in acetylation-dependent apoptotic REs with GG in positions two and three and eight and nine of the ½-site (34). The loop L1 flexibility induced by the S139F mutation promotes an increase in transcription above the WT baseline seen for the GGGCA full-site sequence, as well as for KILLER and FAS REs that have 85 and 65% sequence identity, respectively, to the GGGCA full-site RE. Instead, the lower DNA-binding affinity seen for the GAACA sequences for the S139F DBD and for the WT ΔNp73δ isoform might explain the reduced transactivation in yeast and mammalian cell reporter assays for the P21-5′ and GADD45 REs, both of which have a 0/4 RY score, points in the same direction (Supplementary Table S1 and Figure 7). Overall, the increased flexibility of loop L1, the lack of DNA recognition by K138 and the increased tetramerization surface of S139F seem to be correlated with an increase in transactivation levels, particularly for GG-containing REs that in the WT keep loop L1 bound to DNA.

### An evolved RE-code underlies p53 family promoter selectivity and could be tailored to stress-dependent response programs

The p73 S139F and p53 S121F (p63 S150F was not examined) showed increased capacity to induce several apoptotic genes belonging to the extrinsic as well as intrinsic pathways, with the exception of PUMA. Conversely, they showed reduced capacity to activate both the cell cycle arrest p21 gene and especially the MDM2 gene at mRNA and protein levels (Figure 3) (36,78). Looking at the functional classification of the genes associated with the REs examined in the transactivation assays, we noted that S139F had enhanced activity for seven of eight REs of apoptotic genes (Supplementary Table S1). To explore this correlation further, we took advantage of a recently compiled list of validated p53 target genes (22). Using stringent RE pattern search criteria (42) as well as p53 ChIP-seq data (81), 58 REs derived from 54 genes were considered, grouped according to the RY signatures and interrogated using the functional clustering enrichment tool within DAVID (http://david.abcc.ncifcrf.gov/). Notably, the enrichment for apoptosis function based on gene ontology was four orders of magnitudes higher for the group of REs from genes predicted to be super-transactivated by p73 S139F (highest *P*-values in the cluster = 2e^−^^9^). Only two genes, PUMA and CASP1, contain p53 REs to which S139F is expected to show reduced transactivation potential. Of these, the PUMA RE has moderate transactivation potential based on its sequence features. Interestingly, the discovery of an insulator-type RNA-polymerase II stop site in proximity to exon 3 led to the proposal (82) that there is a post-transcriptional initiation mechanism for the regulation of PUMA expression, suggesting that in this case fine tuning of p53 affinity for the promoter may not be critical.

Although reduced DNA-binding affinity has been previously noted for apoptotic p53 REs (13,22), our study indicates that sequence features affecting torsional flexibility and the cooperative nature of p53 tetramer binding to REs have been selected in evolution to contribute to *in vivo* transcriptional selectivity (32,59,61,83). For example, the p53 DBD differs from p73 DBD in the capacity to form an intra-dimer salt bridge, and p53 mutants at residues establishing salt bridge interactions within the DBD were shown to exhibit enhanced cooperativity and to induce higher levels of apoptosis when expressed in human cells (32). Our DNA-binding studies for p73 S139F, published DNA-binding results with p53 S121F (36,78,80) along with our crystal structure of p73 S139F bound to DNA indicate that conformational changes in the L1 loop can also greatly impact sequence-specific target recognition, impact on cooperative binding to DNA and result in higher induction of apoptosis in cells.

Notably, a physiologically occurring post-translational change, namely, the acetylation of K120, within p53 DBD, was also associated with higher apoptotic potential, relating to changes in DNA sequence-specific versus non-specific discrimination (76,84). To date, an equivalent change has not been reported for p73 or p63. Acetylation of K120 was proposed to facilitate L1 loop conformational changes. Modelling S121F with a rotamer library supported the view that this mutation could also favour a similar shift in the L1 loop conformation. Further the mutant showed faster off rates with an RY = 0/4 consensus RE and reduced transactivation activity (30).

Hence, we propose the selection during evolution of an RE sequence code within target promoters, which coupled to stress-induced post-translational modifications, can affect intrinsic conformational flexibility of p53 proteins. The combination of these factors can alter DNA-binding affinity and cooperative binding so as to modulate *in vivo* selectivity, favouring the activation of apoptotic targets.

## ACCESSION NUMBERS

Crystal structure of p73DBD S139F bound to DNA PDB ID: 4guq.

## SUPPLEMENTARY DATA

Supplementary Data are available at NAR Online, including [85].

## FUNDING

Funding for open access charge: Italian Association for Cancer Research (in part) (Associazione Italiana per la Ricerca sul Cancro), AIRC [#IG9086 to AI and IG#5506 to G.F.] and CIBIO start-up funds; NIEHS intramural research funds (to M.A.R. and D.M.) project [Z01-ES065079 to M.A.R.]; Marie-Curie/Autonomous-Province-of-Trento (to Y.C.) (PAT) cofund grant [#40101712]; Diffraction data were collected at BL7-1 of the Stanford/Stanford Synchrotron Radiation Lightsource supported by the Department of Energy and National Institutes of Health [P41RR001209].

*Conflict of interest statement*. None declared.

## Supplementary Material

Supplementary Data
