# Supplementary material for: Transactivation specificity is conserved among p53 family proteins and depends on a response element sequence code
Source: Nucleic Acids Res. 2013 Jul 26;41(18):8637–53. doi: 10.1093/nar/gkt657 (PMC3794606; doi:10.1093/nar/gkt657)
Supplement: Supplementary Data [file supp_41_18_8637__index.html]

Transactivation specificity is conserved among p53 family proteins and depends on a response element sequence code — Transactivation specificity is conserved among p53 family proteins and depends on a response element sequence code — Supplementary Data 

# Transactivation specificity is conserved among p53 family proteins and depends on a response element sequence code

## 

files

**Files in this Data Supplement:**

- Supplementary Data - pdf file
