# Supplementary material for: Transactivation specificity is conserved among p53 family proteins and depends on a response element sequence code
Source: Nucleic Acids Res. 2013 Jul 26;41(18):8637–53. doi: 10.1093/nar/gkt657 (PMC3794606; doi:10.1093/nar/gkt657)
Supplement: Supplementary Data [file supp_gkt657_nar-00444-x-2013-File009.pdf]

Ciribilli, Monti *et al.*, **Supplementary Figures and Tables.**

Figure S1. Evolutionary conservation of altered-function amino acid positions in p53 family proteins.

A) Sequence alignment of the DNA binding domains of human p53 family proteins. Human protein sequences were obtained from Uniprot Knowledgebase (Release 2010-2011) and aligned with ClustalW at PBIL using default setting (85). Highlighted are amino acids that show identity (red, "*"), strong similarity (green, ":"), weak similarity (blue, ".") or are different (black) according to the alignment software. The position of the altered function p53 mutants S121F, T123A and S240N with the corresponding numbering for TA p73 (S139F, T141A and S260N) and TA p63 (S150F, T152A, S271N) is also indicated. The numbering for the protein alignment refers to the TA p73 gene protein sequence. **B**) Extended list of p53 amino acid changes that exhibited enhanced transcriptional activation and/or altered promoter selectivity (35). The localization of p53 residues in the p53 DNA binding domain structure (55,63), the corresponding p73 and p63 residues and their amino acid similarity are indicated.

Figure S2. Relative transactivation potential of altered function mutants constructed in the TA p73 and TA p63 β isoforms.

The experiment were conducted and elaborated as described for Figure 1. **A)** Transactivation potential with the yeast-based assay of the indicated TA p73β towards the P21-5'-, PUMA-, MDM2-, KILLER-, BAX A+B-, FAS-, GADD45- and AIP1-REs, ordered based on decreasing relative transactivation potential obtained with WT p73β protein. **B)** Four REs were also examined with TA p63β WT and the S150F or S271N mutants. Presented is the mean relative fold of inductions over the empty vector, obtained upon normalization to unit of soluble proteins (light unit/μg protein), and standard deviations of four biological replicates. The moderate *ADH1* promoter was used to express all proteins. **C)** Transactivation phenotype of p73β S139F and S260N alleles is

confirmed at different levels of expression. The transactivation capacity of p73 WT, S139F and S260N (β isoform) was also measured using the inducible *GAL1,10* promoter to achieve variable levels of expression in the yLFM-P21-5', yLFM-BAX-A+B, yLFM-GADD45 and yLFM-AIP1 reporter strains. Presented are the means and standard deviations of four different biological replicates of the luciferase activity obtained after eight hours of culture in 0.016% galactose (moderate expression) or 0.128% galactose (high expression). Relative light units (RLU) were obtained upon normalization to OD_{600} and used to calculate the fold of induction over the empty expression vector (pRS314). The *GAL1,10* expression p73 vectors were constructed using standard cloning starting from the available pTS-p73β, pTS-p73β S139F and pTS-p73β S260N and the pTSG expression vector as recipient (60).

Figure S3. Relative WT and mutant p53 family protein expression in yeast.

A. p73 and p63 proteins are expressed at lower levels in yeast compared with p53. Western Blot was performed to test the p53 family member expression in yeast cells. p53 family protein members fused at the 5'-end with 3 repeats of the HA-tag were expressed under the galactose inducible promoter (*GAL1,10*) and cultured in selective media containing glucose (2% where the expression of the proteins is inhibited) or galactose (1%) to increase the transcription rate of the p53 family members. After 24 hours of growth at 30°C cell were collected and lysed as described in (38). Increasing amount of soluble proteins (25 μg and 50 μg respectively) was loaded; p53 as well as p73 (α and β isoforms were tested) and p63α expression levels were detected with the Y-11 antibody raised against HA tag (Santa Cruz Biotechnology).

B. p73 S139F and p73S260N protein levels are reduced compared to WT p73. Two isogenic yeast reporter strains, yLFM-P21-5' and yLFM-AIP1 were transformed with ADH1-based expression vector for p73 alleles: (1) empty vector, pRS314; (2) WT p73, pTS-HA p73β; (3) S139F, pTS-HA p73β S139F; (4) S260N, pTS-HA p73β S260N. The expression of mutant p73 relative to the WT, calculated from densitometric scanning of immunoreactive bands for p73 and actin loading control, is indicated.

Figure S4. p53 transactivation potential can be influenced by protein concentration.

Transactivation ability of WT p53 allele under the inducible *GAL1,10* promoter in the yLFM-P21-5', yLFM-PUMA, yLFM-KILLER and yLFM-GADD45 reporter strains. The transactivation ability was determined using an inducible expression of p53 protein under the *GAL1,10* promoter (16 hours in 0.002% galactose and 0.016% galactose). Presented are the means and standard deviations of four different biological replicates of the luciferase activity. Relative light units (RLU) were obtained upon normalization to unit of soluble proteins (light unit/μg protein) and used to calculate the fold of induction over empty expression vector (pRS314). For the luciferase assay, cells were treated as previously described in Materials and Methods.

Figure S5. 2Fo-Fc map of the loop L1 region used for model building.

Presented is a representative area of the 2Fo-Fc map we used to model the DBD region surrounding loop L1. The cut-off is 1.5σ. An unbiased omit map showed the backbone density, but it was more discontinuous and difficult to use for model building. Lys138 does not contact the DNA in either monomer. However, due to the flexibility of loop L1, it cannot be discarded that Lys138 could transiently contact the DNA, even though in a manner clearly different than the WT p73.

Figure S1

A

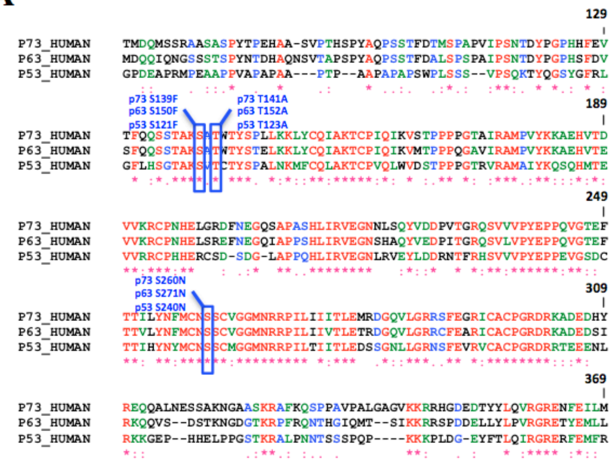

B

p53	ST-AA	Domain	p73	p63	Similarity
H115	R	L1	Q133	Q144	strongly similar
S116	T	L1	S134	S145	identity
S121	F	L1	S139	S150	identity
T123	A	L1	T141	T152	identity
C124	Y or F	L1	W142	W153	different
Q167	R	L2	E185	E196	strongly similar
E171	K	L2	D189	E200	strongly similar
H178	Y	H1	N196	N207	strongly similar
S183	L	L2	R201	R212	different
D184	Y	L2	D202	E213	strongly similar
P191	L	L2	A211	A222	weakly similar
E198	V	S5	E218	E229	identity
S215	T	S7	S235	S246	identity
T231	I	S8	T251	T262	identity
S240	N	L3	S260	S271	identity
V274	A	S10	I294	I305	strongly similar

Figure S2

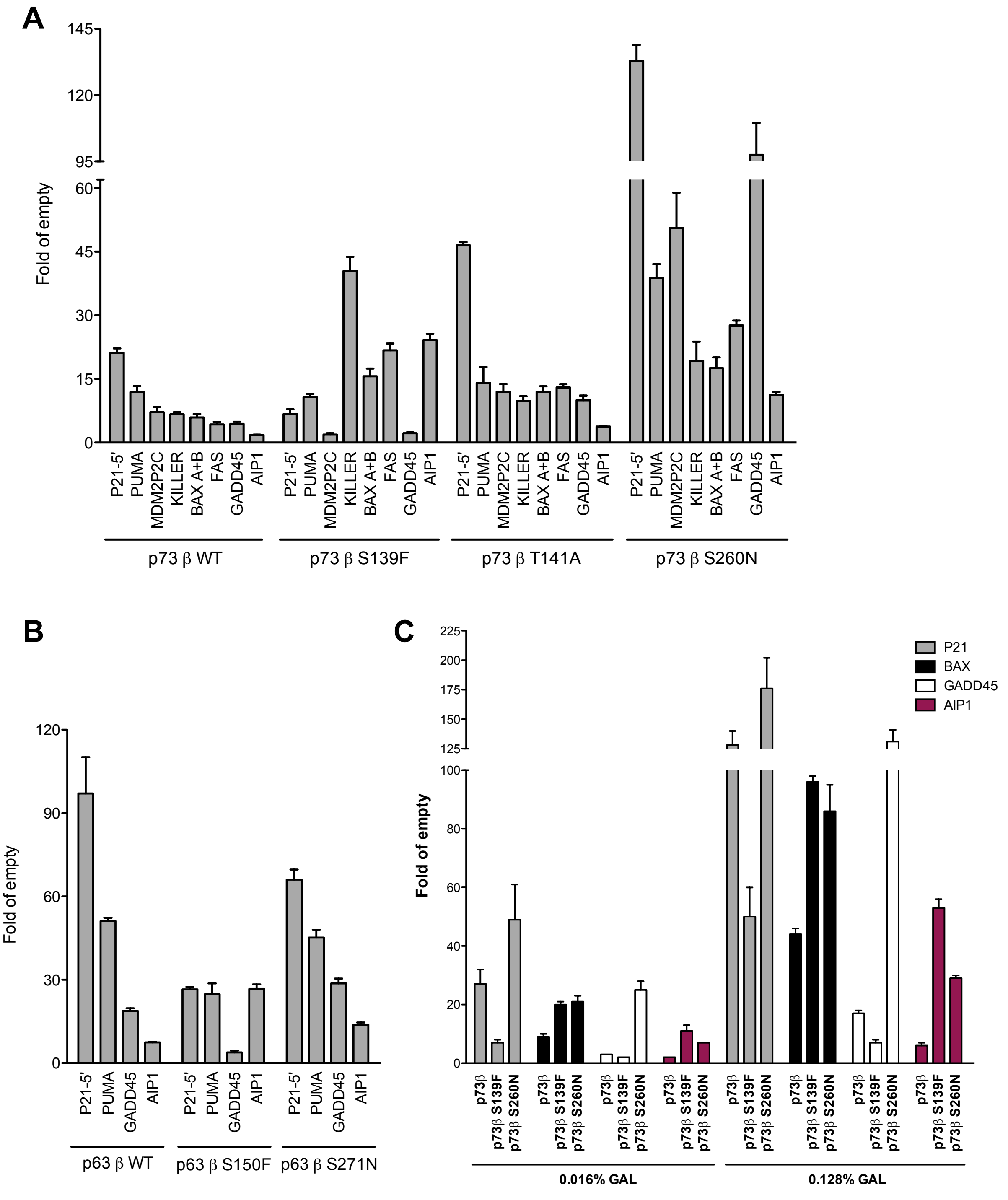
A
145
120
95
60
45
30
15
0
Fold of empty
P21-5'
PUMA
MDM2P2C
KILLER
BAX A+B
FAS
GADD45
AIP1
p73 β WT
p73 β S139F
p73 β T141A
p73 β S260N
B
120
90
60
30
0
Fold of empty
P21-5'
PUMA
GADD45
AIP1
p63 β WT
p63 β S150F
p63 β S271N
C
225
200
175
150
125
100
80
60
40
20
0
Fold of empty
P21
BAX
GADD45
AIP1
p73β
p73β S139F
p73β S260N
0.016% GAL
0.128% GAL

Figure S3

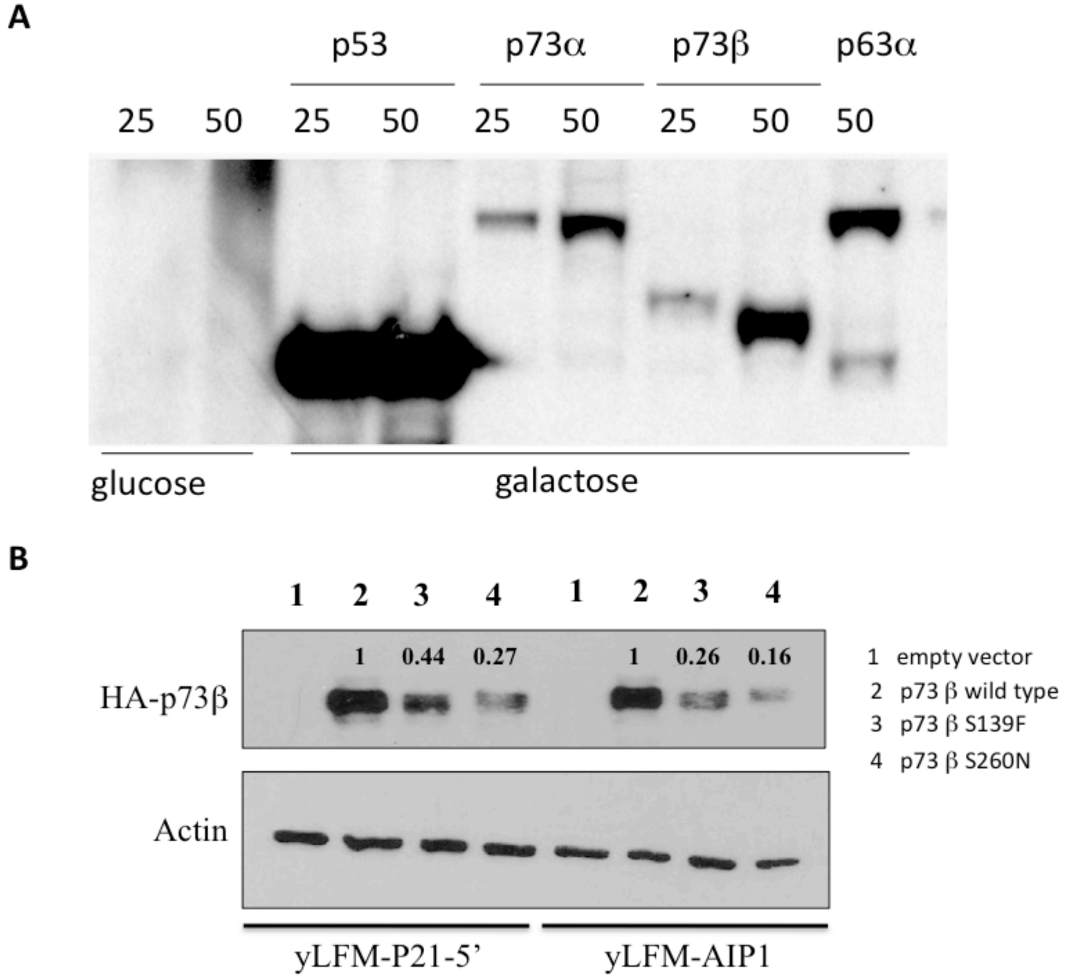
A
p53
p73α
p73β
p63α
25 50 25 50 25 50 25 50 50
glucose
galactose
B
1 2 3 4 1 2 3 4
1 0.44 0.27
1 0.26 0.16
HA-p73β
Actin
yLFM-P21-5'
yLFM-AIP1
1 empty vector
2 p73 β wild type
3 p73 β S139F
4 p73 β S260N

Figure S4

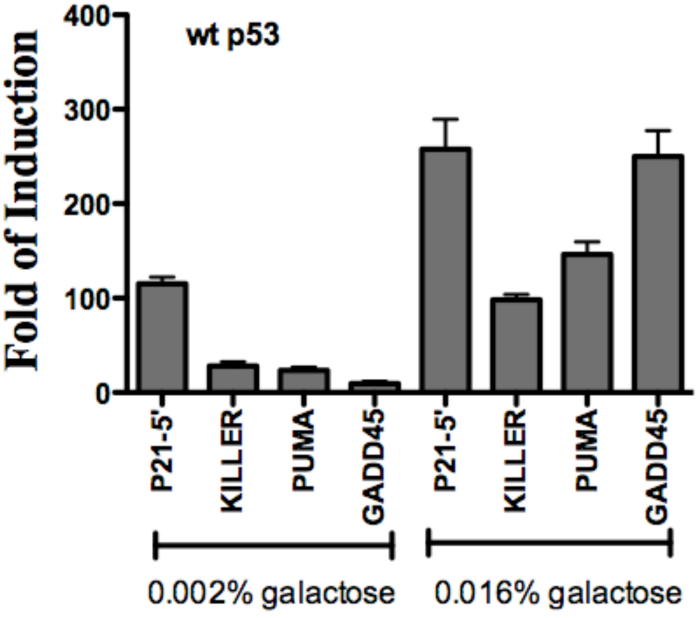

wt p53
Fold of Induction
400
300
200
100
0
P21-5'
KILLER
PUMA
GADD45
P21-5'
KILLER
PUMA
GADD45
0.002% galactose
0.016% galactose

Figure S5

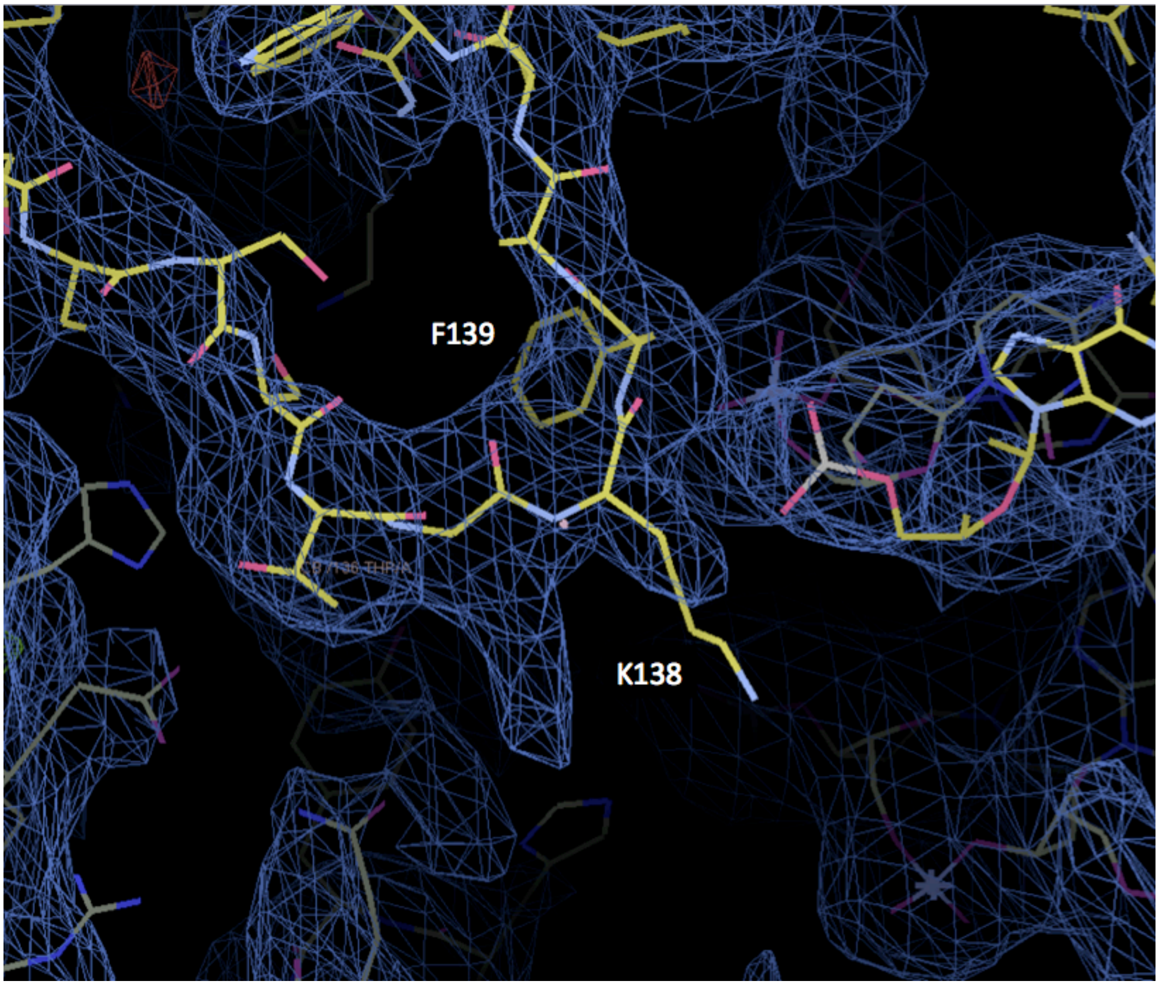

Table S1. List of the REs tested with the yeast-based transactivation assay.

Summary of p73 WT, S139F and S260N alleles (β isoform) transactivation capacity towards 48 p53 REs measured using the yeast functional assay. For all the experiments the average RLUs were normalized by OD_{600} (38) and results obtained with an empty expression vector (pRS314) were used to calculate the fold of induction. p73 alleles were expressed at moderate levels under the *ADH1* constitutive promoter. In the table, REs are ordered based on decreasing transactivation capacity for p73 S139F relative to WT p73 and grouped according to the RE organization (full-, ¾- and ½-sites).

A scheme of the consensus p53 RE is presented. Boxes in blue and roman numbers mark each dinucleotide motif that is considered in the RY score, following the formula shown below the RE sequence. The RY score and the position of the dinucleotide motifs are summarized in columns 5a and 5b, respectively. Green boxes mark the WW motifs (see column 5c).

The various columns of the table report: 1) the name of the p53 RE; 2) the functional classification of the gene associated with the RE as provided in gene ontology (A=apoptosis; C= cell cycle regulator; D= DNA repair; G= angiogenesis; M= metabolism; O= other functions; R= p53 regulation; S= synthetic RE); 3) the sequence of the RE; 4) the relative transactivation capacities calculated as: a) fold of induction over the empty vector for p73 WT, b) the transactivation ratio between S139F and WT and c) the transactivation ratio between S260N and WT; 5) the RE code obtained from Weblogos including a) the number of RY signatures, b) the position of the RYs within the consensus and c) the WW sequence for each half-site (see main text for details);

The p53 REs that were used to compile the WebLogo comprise REs where p73 S139F exhibited transactivation ratio relative to WT greater than 1.5 or lower than 0.67 cut-off. The two groups are highlighted in light blue and orange, respectively. Highlighted in gray are REs that were not functional for any p73 alleles. To develop the logos presented in Figure 3 only full-site canonical REs were included (see main text for details). Besides ½-sites and ¾-sites, 14-3-3σ RE was also excluded because of the non-canonical CWwwWG sequence. MDM2-P2C and BAX A+B REs contain two complete full-sites that were considered separately for the logos.

Table S1

I II III IV

R_1 R_2R_3 C_4 W_5W_6 G_7 Y_8Y_9 Y_{10} – R_{11} $R_{12}R_{13}$ C_{14} $W_{15}W_{16}$ G_{17} $Y_{18}Y_{19}$ Y_{20}

RY score: numerator = $\sum (RR=GG + YY=CC)_{I\text{-}IV}$; denominator = $\sum (RR + YY)$

p53-RE	Function	RE SEQUENCE	TRANSACTIVATION RATIO			RE CODE		
**Canonical site**		RRRCWWGYYY-RRRCWWGYYY	β WT/empty	β S139F/β WT	β S260N/β WT	RY	Position	WW - WW
NOXA	A	AGGCTTGCCC-CGGCAAGTTG	2.2	84.3	14.7	3/4	I - II - III	TT – AA
CYCLIN G	A	AGGCTTGCCC-GGGCAGGTCT	3.6	30.6	9.5	3/4	I - II - III	TT – AG
miR-202	O	GGGCATGTCC-TGGCAAGCCT	8.7	27.8	14.6	3/4	I - III - IV	AT – AA
RGC	S	GGACTTGCCT-GGCCTTGCCT	3.6	27.1	11.4	2/4	II - IV	TT – TT
miR-198	O	AGGCAAGCTT-CAACAAGCCG	1.6	23.9	10.2	2/4	I - IV	AA – AA
AIP1	A	TCTCTTGCCC-GGGCTTGTCG	2.7	12.4	4.1	2/4	II - III	TT – TT
P21-3'	C	GAAGAAGACT-GGGCATGTCT	7.0	12.1	5.7	1/4	III	AA – AT
miR-34a-RE1	A	GGGCTTGCCT-GGGCTTGTTC	14.8	9.8	4.4	3/4	I - II - III	TT – TT
KILLER	A	GGGCATGTCC-GGGCAAGACG	6.4	9.6	5.6	2/4	I - III	AT – AA
XPC	R	GGGCATGGTG-GCACATGCCT	1.6	9.2	7.5	2/4	I - IV	AT – AT
miR-151	O	TGGCTTGTTT-TGGCAAGTTC	1.2	6.0	6.5	2/4	I - III	TT – AA
CON L	S	GGGCATGCTC-GGGCATGCTC	12.9	5.0	3.5	2/4	I - III	AT – AT
BAX A+B	A	TCACAAGTTAgAGACAAGCCT	11.5	4.4	2.5	1/4	IV	AA – AA
		AGACAAGCCT-GGGCGTGGGC				2/4	II - III	AA – GT
CON C	S	GGGCAAGTCT-GGGCAAGTCT	65.3	4.0	2.9	2/4	I - III	AA – AA
hFAS	A	TGGCTTGTCA-GGGCTTGTCC	7.6	3.6	5.0	2/4	I - III	TT – TT
PAI	G	ACACATGCCT-CAGCAAGTCC	1.6	2.9	6.4	1/4	II	AT – AA
SEI 1	O	GGGCTTGAGG-GCGCATGCCC	1.0	2.8	3.2	2/4	I - IV	TT – AT
PCNA	R	GAACAAGTCC-GGGCATATGT	1.1	2.8	8.9	1/4	III	AA – AT
R2	D	TGACATGCCC-AGGCATGTCT	36.1	2.5	2.5	2/4	II - III	AT – AT
CON A	S	GGGCATGTCC-GGGCATGTCC	26.6	1.9	1.7	2/4	I - III	AT – AT
MMP2	G	AGACAAGCCT-GAACTTGTCT	38.3	1.8	3.2	1/4	II	AA – TT
14-3-3σ	C	TAGCATTAGCCC-AGACATGTCC	1.2	1.7	4.3	1/4	II	AT – AT
BAI	G	TGGCTGCCT--GGACATGTTC	1.2	1.5	5.9	1/4	I	TG – AT
CATHEPSIN D	G	AGGCAGGGCC-GGGCTGACCC	1.1	1.1	1.0	3/4	I - III - IV	AG – TG
MSH2	R	GACCTAGGCGcAGGCATGCGC	1.2	1.1	0.9	1/4	III	TA – AT
mFAS	A	GGGCATGTAC-AAACATGTCA	44.4	1.1	3.0	1/4	I	AT – AT
TIGAR	M	AGACATGTCCacAGACTTGTCT	0.9	0.9	1.0	0/4	-	AT – TT
miR-221	O	GAACATGCAT-GCACATGTTT	1.7	0.8	17.7	0/4	-	AT – AT
PUMA	A	CTGCAAGTCC-TGACTTGTCC	24.3	0.6	2.7	0/4	-	AA – TT
GADD45	C	GAACATGTCT-AAGCATGCTG	7.0	0.4	7.7	0/4	-	AT – AT
CON E	S	GAGCATGTCC-GAGCATGTCC	13.3	0.3	3.4	0/4	-	AT – AT
P21-5'	C	CAACATGTTG-GGACATGTTC	36.9	0.3	1.9	0/4	-	AT – AT
p21 S2	C	GAACAGGTCC-CAACAGGTTG	5.7	0.2	6.2	0/4	-	AG – AG
MDM2-P2C	R	GGTCAAGTTG-GGACACGTCC	16.8	0.2	3.3	0/4	-	AA – AC
		GAGCTAAGTCcTGACATGTCT				0/4	-	TA – AT
CON S	S	GAACATGTTC-GAACATGTTC	85.3	0.1	1.3	0/4	-	AT – AT
PA26	C	GGACAAGTCT-CAACAAGTTC	32.4	0.1	3.0	0/4	-	AA – AA

**3/4 site**		XXXXX-RRRCWWGYYY-XXXXX						
P21-3' complete	C	GGGCATGTCT-GGGCAC	2.3	30.4	12.7	2/3	I - III	AT – AC
CON J	S	GGGCATGTCC-GGGCAC	2.0	18.8	8.3	2/3	I - III	AT – AC
CON K	S	GGGCATGTCC-TGTTTTGTCC	1.1	1.2	3.1	1/3	I	AT – TT
miR-10b	O	CTGTCT-GAACAAGTCG	4.9	0.3	16.6	0/3	-	CT – AA
**1/2 site**		RRRCWWGYYY						
CON D 1/2	S	GGGCATGCCC	0.8	22.5	1.7	2/2	I - II	AT
CON O 1/2	S	GGGCAAGCCC	1.7	9.7	1.0	2/2	I - II	AA
CON G 1/2	S	GGGCATGTCT	0.6	2.9	1.5	1/2	I	AT
miR-34a-RE2	A	GAGCATGCCC	1.0	1.8	1.2	1/2	II	AT
CON H 1/2	S	GGGCTTGTCC	0.9	1.3	1.0	1/2	I	TT
CON I 1/2	S	GGGCAAGTCC	1.1	1.3	1.1	1/2	I	AA
CON E 1/2	S	GAGCATGTCC	0.9	1.2	1.1	0/2	-	AT
miR-34a-RE3	A	AGACTTGCCT	1.4	0.9	0.6	1/2	II	TT

Supplementary Table 2. Data collection and refinement statistics

CRYSTALS	Ser139Phe p73 DBD Structure P73DBD/20mer
**PDB ID**	**4guq**
**DNA sequence**	**5'-GAACATGTTCGAACATGTTC -3'**
**DATA COLLECTION**	
**Wavelength (Å)**	1.23
**Space group**	$P6_1$
**Cell dimensions**	
a, *b*, *c* (Å)	172.49, 172.49, 34.09
α, β, γ (°)	90.00, 90.00, 120.00
**Resolution (Å)**	50.0 – 3.7 (3.8 – 3.7)
R_{sym} **or** R_{merge}	10.5 (39.5)
$I / \sigma I$	5.4 (1.9)
**Completeness (%)**	96.7 (95.6)
**Redundancy**	1.9 (1.8)
**REFINEMENT**	
**Resolution (Å)**	100.0 – 3.7
**Number of reflections**	6,475
R_{work} / R_{free}	30.3 / 31.6
**Molecules in asymmetric unit**	
**Protein (198 aa) / dsDNA (10 bp)**	2 / 1
**No. atoms**	3,547
Protein	3,158 (NCS: 1579)
DNA / Zn^{2+} ion	412 / 2
***B*-factors**	81.6
Protein	81.4
DNA / Zn^{2+} ion	83.0 / 85.9
**r.m.s. deviations**	
Bond lengths (Å)	0.019
Bond angles (°)	2.0
Dihedral angles (°)	24.5
**Ramachandran Plot (%)**	
Resd. in most favored region	89.1
Resd. in additional allowed region	10.9
Resd. in generously allowed region	0.0
Resd. in disallowed region	0.0
G-factor	-0.22

Values in parentheses are for highest-resolution shell. The data included in the Rfree set (2.4%) was excluded from refinement. Geometry of the final model was evaluated with PROCHECK. PDB, Protein Data Bank; r.m.s., root mean square. Resd, residues.
